# Computational Methods for the Discovery and Optimization of TAAR1 and TAAR5 Ligands

**DOI:** 10.3390/ijms25158226

**Published:** 2024-07-27

**Authors:** Naomi Scarano, Stefano Espinoza, Chiara Brullo, Elena Cichero

**Affiliations:** 1 Department of Pharmacy, Section of Medicinal Chemistry, School of Medical and Pharmaceutical Sciences, University of Genoa, Viale Benedetto XV, 3, 16132 Genoa, Italy; naomi.scarano@edu.unige.it (N.S.); chiara.brullo@unige.it (C.B.); 2 Department of Health Sciences and Research Center on Autoimmune and Allergic Diseases (CAAD), University of Piemonte Orientale (UPO), 28100 Novara, Italy; stefano.espinoza@uniupo.it; 3 Central RNA Laboratory, Istituto Italiano di Tecnologia (IIT), 16152 Genova, Italy

**Keywords:** TAAR1, TAAR5, molecular modeling, docking, mutagenesis, drug design

## Abstract

G-protein-coupled receptors (GPCRs) represent a family of druggable targets when treating several diseases and continue to be a leading part of the drug discovery process. Trace amine-associated receptors (TAARs) are GPCRs involved in many physiological functions with TAAR1 having important roles within the central nervous system (CNS). By using homology modeling methods, the responsiveness of TAAR1 to endogenous and synthetic ligands has been explored. In addition, the discovery of different chemo-types as selective murine and/or human TAAR1 ligands has helped in the understanding of the species-specificity preferences. The availability of TAAR1–ligand complexes sheds light on how different ligands bind TAAR1. TAAR5 is considered an olfactory receptor but has specific involvement in some brain functions. In this case, the drug discovery effort has been limited. Here, we review the successful computational efforts developed in the search for novel TAAR1 and TAAR5 ligands. A specific focus on applying structure-based and/or ligand-based methods has been done. We also give a perspective of the experimental data available to guide the future drug design of new ligands, probing species-specificity preferences towards more selective ligands. Hints for applying repositioning approaches are also discussed.

## 1. Introduction

Trace amines (TA), a group of endogenous compounds found at low levels in both peripheral and brain tissues of vertebrates, notably mammals, encompass classic examples like **β-phenylethylamine (β-PEA**), **p-tyramine**, **tryptamine**, and **octopamine** [1]. Initially considered inert byproducts of endogenous monoamines, such as dopamine and serotonin, their significance was reevaluated with the discovery of the trace amine-associated receptor (TAAR) family [2,3]. TAARs comprise nine subfamilies encoded by distinct genes and pseudogenes across species, including six genes (TAAR1, TAAR2, TAAR5, TAAR6, TAAR8, and TAAR9) and three pseudogenes (TAAR3, TAAR4, and TAAR7) in humans, and fifteen functional genes in mice [1,4]. Except for TAAR1, other TAARs are predominantly expressed in the olfactory epithelium, forming a unique class of olfactory receptors sensitive to volatile amines linked to innate behaviors [5,6]. However, recent evidence has demonstrated the expression of different TAARs outside the olfactory systems, including specific brain regions [7,8,9].

Among TAARs, TAAR1 has received the most attention, responding not only to trace amines but also to amphetamines and other psychotropic compounds [2,3]. It is expressed at low levels in the brain and periphery. In the central nervous system, TAAR1 is present in regions that are important for the regulation of monoamine systems, such as the ventral tegmental area, the *substantia nigra*, the dorsal raphe, the prefrontal cortex, and the amygdala [3,10,11]. It regulates the dopamine system, impacting D2 dopamine receptor activity and dopaminergic neuron firing [10,12,13,14,15]. TAAR1 knockout (TAAR1-KO) mice display heightened behavioral and neurochemical responses to dopaminergic compounds, presenting TAAR1 as a promising pharmacotherapeutic target for psychiatric disorders [16]. Recent clinical trials indicate the potential use of TAAR1 agonists for schizophrenia treatment, offering a novel mechanism independent of D2 dopamine receptor blockade [17].

TAAR5, another receptor of the TAAR family, shares a similar brain expression profile with TAAR1. Found in limbic regions such as the amygdala, entorhinal cortex, and *nucleus accumbens*, TAAR5 modulates emotional behavior and serotonin system function [7]. TAAR5 knockout (TAAR5-KO) mice exhibit anxiolytic and antidepressant-like behaviors, along with alterations in serotonin levels and enhanced 5-HT_1A_ serotonin receptor function (5-HT_1A_R). Furthermore, TAAR5 influences dopamine levels and adult neurogenesis and is involved in sensorimotor functions and cognitive processes like attention and motivation [18,19,20,21]. This evidence positions TAAR5 as a promising drug target for mood disorders and cognitive impairment.

The development of compounds targeting TAAR1 has been extensive in the last 15 years, with Hoffman-La Roche acting as a pioneer in characterizing the first potent and selective TAAR1 full, partial agonists, and antagonists. Another company, Sunovion Pharmaceuticals (now Sumitomo Pharma), developed a TAAR1/5-HT_1A_R agonist, **SEP-363856** (**Ulotaront**) [22], which evidenced promising results in a phase II clinical trial for schizophrenia [23,24,25,26]. These data boosted the research on discovering new TAAR1 ligands and the effort to understand the mechanism of how endogenous and synthetic TAAR1 agonists bind to the receptor. The pharmacology of TAAR5, less studied than TAAR1, is still in its infancy and only a few ligands have been described. This review describes the work conducted so far into the computational methods used to discover ligands for these two members of the TAAR family, by giving an update of the comprehension of the mechanisms of ligand–receptor interactions. A perspective of the experimental data available and of the viability of repositioning strategies has also been detailed.

## 2. Molecular Modeling Studies in the Discovery and Optimization of TAAR1/5 Ligands

### 2.1. Computational Methods Exploring mTAAR1 Ligands

**Figure 1:**
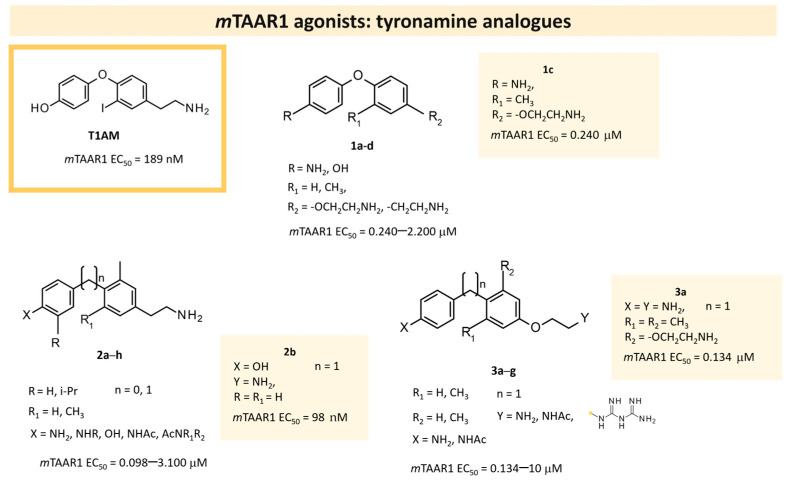
Scheme of three series of T1AM analogues (**1**–**3**) [27,28] developed as TAAR1 agonists. The most effective compounds of the series have been reported.

In search for novel agonists active on the murine orthologue of TAAR1, Chiellini et al. rationally designed a small series of thyronamine analogs, which were tested in vitro [27]. The final aim of their study was to understand the molecular basis of TAAR1 activation by designing thyronamine derivatives (**1**) as synthetic analogues of the endogenous TAAR1 agonist **T1AM** (Figure 1).

In detail, the authors replaced the oxygen atom tethered to the two aromatic rings of the endogenous ligand, with an isosteric methylene linkage. The OH group was maintained or replaced with the NH_2_ group, which retains the same H-bonding donor and acceptor capabilities of the OH group. Finally, the amine-ethyl portion was considered or changed in a terminal amine-ethoxy function (see Figure 1).

Following biological assays, the compounds **1a–d** were highlighted, the most potent of them, **1c**, exhibiting a comparable EC_50_ to **T1AM** (EC_50_ = 240 nM; **T1AM** EC_50_ = 189 nM) (Table 1, entry 1). 

To investigate the binding mode of the newly synthetized compounds at the receptor binding site, a docking procedure was performed in a homology model (HM) of the *m*TAAR1, built according to a ligand-based homology modeling procedure [29]. In particular, the HM was developed using the X-Ray of β_2_-adrenoreceptor (β_2_-ADR) in complex with an irreversible agonist as a template (PDB ID = 3PDS) [30], and the reference compound included in the ligand-based HM calculations was **T1AM**. The construction of the receptor HM employed the MOE software (MOE2013) [31]. The procedure included the alignment of the target sequence to the template by means of the BLOSUM62 matrix, followed by a loop search to rebuild the missing portions. The most promising model was selected according to the best packing quality function. After a minimization step, the quality of the obtained model was evaluated through comparison with the Ramachandran plot. The docking of the candidates was performed via the Surflex docking module implemented in Sybyl-X1.0 [32], and the best-scored poses were selected for the ligand/receptor energy minimization. Such poses were further submitted to ten runs of docking with the MOE-Dock genetic algorithm. The poses with best scores and lowest RMSD with respect to the output of the minimization were selected as the most stable poses. The position of the binding site was derived through a comparison with the template complex.

The docking analysis provided important clues as to the interaction mode of the tested compounds. In detail, the most potent compound, **1c**, exhibited two H-bonds to D102 and Y291, in addition to two cation–π interactions (the first between the ligand protonated amine and residue Y287, the second between residue R86 and the ligand aniline ring). Moreover, a π–π stacking interaction was observed between the ligand aminoethyloxy-phenyl moiety and residue Y287. The study also pointed out some structural modifications tolerated on the thyronamine scaffold: the replacement of the phenol hydroxyl with an amino group, the increase in the distance between the charged amine and the aromatic ring by inserting an oxygen bridge, and the replacement of the 3-iodo substituent with an alkyl group. 

On this basis, the lead compound (**1c**) was further optimized in a following study [28] involving the docking-based drug design, synthesis, and in vitro evaluation of fourteen analogs (**2**, **3**). The introduced modifications were intended to restore a H-bond with R82 present for **T1AM** and not for compound **1c**, with the introduction in **2** of the ethylamine chain in place of oxo-ethylamino featured by the previous hit **1c** (Figure 1). In addition, the introduction of small alkyl substituents (Me, i-Pr) on both the outer and inner rings, or the removal of the methylene bridge, have been taken into account. This kind of approach has been managed both in the **2** series and in compounds **3**, as highly related **1c** analogues (Figure 1).

In vitro tests of compounds **2**, **3** were performed, and most of them exhibited ameliorated activity at *m*TAAR1 (up to EC_50_ = 98 nM, for compound **2b**) (Table 1, entry 2). In particular, the replacement of the oxy-ethylamino sidechain with the ethylamino one proved to be advantageous, as well as the concomitant replacement of the amino group of the outer ring with the hydroxyl moiety, as shown by **2b** (Figure 1). In addition, the presence of small alkyl substituents onto the phenyl ring tethered to the terminal chain was effective, making most of the derivatives of **2**, **3** more potent than compounds **1** previously.

The docking of the newly synthesized analogues allowed for the rationalization of the obtained results. Surprisingly, the orientation of the two most potent compounds (**2a** and **2b**) was reversed with respect to the first series of thyronamine analogs; however, they did exhibit a favorable network of interaction. In particular, compounds **2a** and **2b** formed a H-bond with D102 with the aniline moiety, while their protonated amine interacted via H-bonds with T83 and D284. However, both of them were selected for further in vivo investigation to ascertain their ability to modulate plasma glucose level. The docking procedure employed the previously obtained HM of *m*TAAR1 and was performed with the Surflex docking module implemented in Sybyl-X1.0. The top-scored poses were submitted to ligand/protein energy minimization by means of the MOE software.

### 2.2. Computational Methods Exploring hTAAR1 Ligands

The first study devoted to the search for *h*TAAR1 ligands involved a computer-aided drug discovery campaign applying an in silico virtual screening (VS) strategy [33]. In detail, a few hundred compounds previously reported as 5-HT_1A_R and/or α_1_-adrenoreceptor (α_1_-ADR) agonists were evaluated via molecular docking calculations [34,35,36,37,38,39]. The exploited *h*TAAR1 structural model was built via homology modeling taking as its template the X-Ray of the human β_2_-ADR in complex with a known agonist (PDB code: 3PDS) [30]. This calculation was achieved by applying MOE software [31]. Following this, docking studies were performed using the Surflex docking tool in the SybylX1.0 software [32]. The binding site in the *h*TAAR1 receptor was defined considering a range of 9Å around the key residue D103. The putative docking mode of **RO5166017**, **β-PEA**, **T1AM** (taken as reference TAAR1 agonists), and **EPPTB** (taken as reference TAAR1 antagonist) [40] was explored and compared with those of the aforementioned GPCR ligands. 

**Figure 2:**
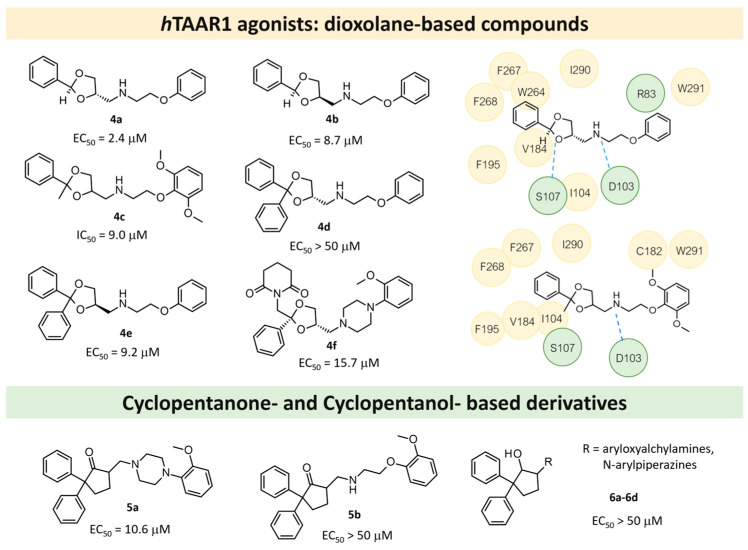
Scheme of the GPCR-targeting compounds **4**–**6** [34,35,36,37,38,39], screened as *h*TAAR1 ligands [33]. The ligplot of the putative docking mode related to **4a** and **4c** have been reported. The most important polar and hydrophobic residues are reported in green and light orange, respectively.

The scouted library comprehended thirty different scaffolds combined with various substitutions and was screened against a previously published HM of the target [40]. The compounds included a series of aryloxyalchylamines and N1-arylpiperazines featuring 1,3-dioxolane-, 1,3-oxathiolane-, 1,3-dithiolane-, spiro-dioxolane-, 1,4-dioxane-, tetrahydrofuran-, cyclopentanone-, and cyclopentanol-based substituents. Among them, compounds **4**–**6** (Figure 2) have been deeply investigated in silico and then evaluated via biological assays.

The corresponding docking analysis revealed the presence of a common interaction pattern, constituted by a H-bond to a D103 sidechain, and π–π stacking with residues W264, F267, and F268 (see Figure 2). This information, together with a similar analysis carried out on **T1AM** and EPPTB as reference *h*TAAR1 agonist and antagonist, guided the compound selection for in vitro tests. In the initial screening phase, seven compounds displayed some activity as a TAAR1 agonist with a maximum effect (Emax), compared with the standard TAAR1 agonist **β-PEA** (EC_50_ = 138 nM), spanning from 40 to 83%. For the most promising compounds, dose response has been calculated revealing **4a** as the more potent in mediating cAMP production by TAAR1. This piece of information allowed for a preliminary exploration of the structure–activity relationship (SAR) within this series of compounds.

The dioxolane-based compounds **4**, bearing a flexible amino group tethered to the terminal phenoxy, was more effective than those featuring the piperazine substituent. On the contrary, the pentanone-(**5**) and the pentanol-(**6**) based compounds were mildly active or inactive, respectively. 

While compounds **4a**,**b** and **4e**,**f** were characterized by *h*TAAR1 agonist activity, compound **4c** proved to be antagonist. As a result, six molecules bearing a dioxolane/cyclopentanone scaffold displayed bioactivity towards the target: in particular, five agonists (**4a**,**b**, **4e**,**f**, and **5a**, *h*TAAR1 EC_50_ = 2.4–15.7 μM) and one antagonist compound (**4c**, *h*TAAR1 EC_50_ = EC_50_ = 9 μM) were individuated. The most interesting agonist, **4a**, and antagonist, **4c**, proposed have been reported in Table 2 (entry 1). 

**Figure 3:**
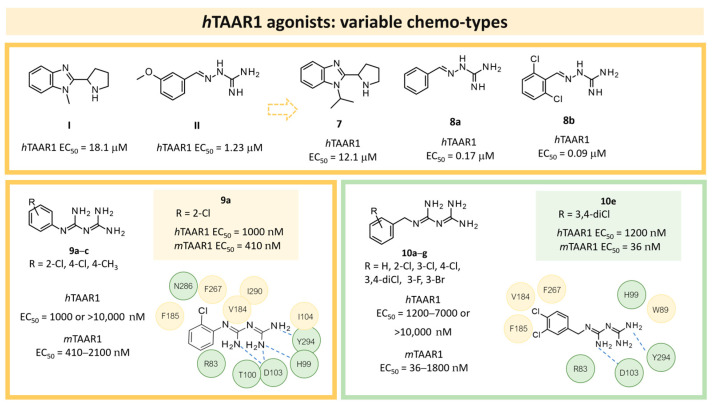
Scheme of the TAAR1 ligands **I**, **II**, **7**, **8** [41], and **9**, **10** [42]. The ligplot of the putative docking mode featured by **9a** and **10e** have been reported. The most important polar and hydrophobic residues are shown in green and orange, respectively.

One year later, Lam et al. published a similar study, performing a large-scale VS of more than 3 billion compounds, comprehending both fragment-like and lead-like compounds and referring to known TAAR1 ligands such as **I** and **II** (Figure 3) [41]. 

A set of 63 known TAAR1 ligands together with 161,000 commercially available compounds were docked to 200 HMs using the DOCK3.6 software [51]. It should be noted that II was also identified as a partial agonist, representing an interesting scaffold for the development of agonist and antagonist series. In detail, the VS was carried out based on an HM of *h*TAAR1 built on the X-Ray structure of the human β_2_ adrenergic receptor (β_2_-ADR), in the presence of the partial inverse agonist carazolol (PBD code = 2RH1) [52]: several HMs were obtained; their screening performance was evaluated via the Receiver Operating Characteristic-Area Under the Curve (ROC-AUC) measure. The best-performing system was selected for the aforementioned VS of two ZINC libraries (a fragment-like library of 0.357 million compounds, and a lead-like library containing 2.7 million molecules). Among the top-scored compounds, forty-two molecules were selected for in vitro tests. Based on the structure-based studies, all the selected compounds were predicted to share important pharmacophore features with known active compounds, such as the capability to form a salt bridge with D103 and the presence of an aromatic moiety protruding towards TM5.

Following in vitro tests, nine TAAR1 agonists were identified, three of them being active in the low μM, such as compounds **7**, **8a**, and **8b** (Figure 3). In Table 2 (entry 2), the chemical structure of **8b** (**Guanabenz**) as a reference-screened compound is reported.

In 2017, Cichero et al. reported a computationally driven study on *h*TAAR1, in which several HMs were compared to guide the design of modulators exploring species-specificity profiles [42]. The most potent *h*TAAR1 agonist identified in this study is reported in Table 2 (**9a**; entry 3).

In detail, the previously published HMs of *h*TAAR1 [40], *m*TAAR1 [27], and *h/m*TAAR5 [42] were analyzed, as built on the same template, namely the X-Ray of β_2_-ADR in complex with a covalently bound agonist (PDB ID = 3PDS) [30]. The putative docking mode of the endogenous ligand **T1AM** was calculated, relying on flexible docking studies using the Surflex docking module implemented in Sybyl-X1.0. According to this analysis, a H-bond to D103 was confirmed to be key for the TAAR1 agonist activity. Scaffold rigidity together with suitable H-bond features emerged as important properties for the design of selective TAAR1 ligands over TAAR5. Moreover, it was noticed that the presence of the phenol moiety in **T1AM** promoted promiscuity between TAAR1 and TAAR5, through the formation of an additional H-bond. Based on the above, compounds **9** and **10** were designed (Figure 3), including a biguanide moiety to meet the aforementioned rigidity criteria, maintaining a key basic moiety, and at the same time, the phenol group was removed to achieve TAAR1 selectivity over TAAR5 [42]. 

Following in vitro tests at *h/m*TAAR1 and *m*TAAR5, the low-μM to nM activity at *m*TAAR1 in eleven compounds out of twenty-seven was highlighted. Some of them also exhibited activity in *h*TAAR1, but with a 2-fold to 13-fold preference for *m*TAAR1 with respect to *h*TAAR1. All the compounds were inactive as *m*TAAR5 ligands. Subsequent SAR analysis allowed them to individuate features for the design of more potent TAAR1 agonists. In particular, proper hindrance at the *ortho*-*para* positions of the benzyl moiety was observed to increase selectivity over *h*TAAR1 and potency at *h*TAAR1, respectively. 

**Figure 4:**
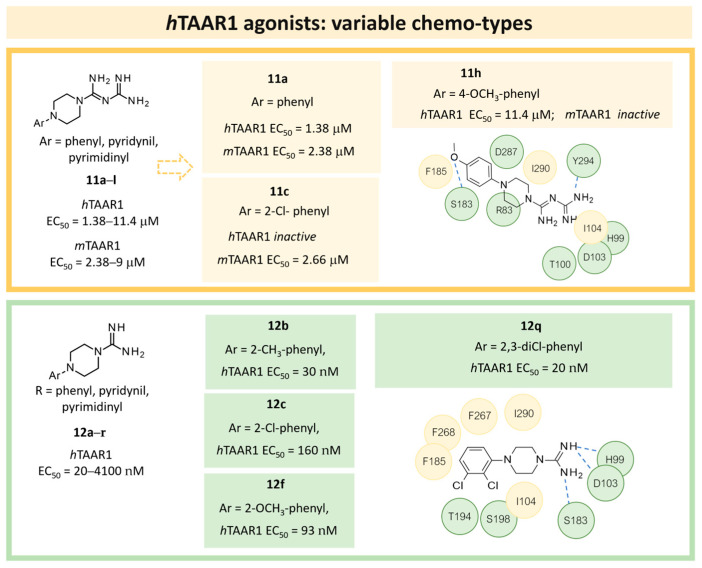
Scheme of the screened TAAR1 ligands **11** [43] and **12** [44]. The ligplot of the putative docking mode featured by **11h** and **12q** have been reported. The most important polar and hydrophobic residues are reported in green and orange, respectively.

In the following years, such a study was pursued to achieve a better understanding of *m/h*TAAR1 selectivity, and at the same time, further optimize the biguanide scaffold [43]. To this aim, two ligand-based QSAR models were developed based on a set of compounds with known activity and species-specificity profiles towards the murine and human orthologues [43], guiding the design of more selective ligands (**11**) (Figure 4). Among them, compound **11h** has been reported as a modest and selective *h*TAAR1 agonist (Table 2, entry 4).

To develop the QSAR models, the collected dataset included thyronamine analogues [27,28], Guanabenz congeners [41], biguanides [42], imidazoles [53], and oxazolines [54]. 

For each model, the molecules were assigned to the training and the test set manually, based on representative criteria of the overall TAAR1 biological activity trend and structural variations. Any compound was explored in terms of geometry and conformation energy by means of the systematic conformational search module included in MOE software [31]. Chemoinformatic and QSAR packages of the same software MOE have been exploited, including molecular descriptors calculation. Afterwards, 302 molecular descriptors (2D and 3D) were obtained, and the resulting matrix was evaluated: the QuaSAR-Contingency and Principal Component Analysis (PCA) tools of MOE were employed for pruning molecular descriptors. The results proposed a few key descriptors to discriminate between murine and human orthologues. In particular, flexibility, as well as the number of polarizable H and positively charged groups, were related to *h*TAAR1 activation, while more rigid and electron-rich groups were predicted to enhance the possibility of activating *m*TAAR1. This information, together with the SAR obtained in the previous study, allowed for the design of the previously cited piperazine-biguanides **11**, which were evaluated in in vitro tests of *h/m*TAAR1 and *m*TAAR5. 

Two selective *m*TAAR1 ligands, such as **11c** (Figure 4), were obtained, and one *h*TAAR1 selective ligand was discovered (**11h**, Figure 4). A docking simulation of these most promising compounds and of the *m/h*TAAR1 promiscuous agonist **11a** explained the observed selectivity. For this analysis, the previously described HMs of *m*TAAR1 [27] and TAAR1 [40] were used.

The choice of a lipophilic electron-withdrawing moiety at the *ortho* position of the aromatic core turns in selective *m*TAAR1 agonists, as shown by **11c**, while the only species-specific *h*TAAR1 agonist, **11h**, exhibited an electron-donor group at the para position of the same ring (see Figure 4). The compound **11h**’s docking pose highlighted one H-bond with S183, thanks to the methoxy substituent, and an additional H-bond with Y294 thanks to the basic moiety. Conversely, the ligand positioning was quite far from the key residue D103. Accordingly, **11h** was a modest but selective *h*TAAR1 agonist.

For the design of dual-acting *m/h*TAAR1 agonists, the introduction of small functions endowed with electron-withdrawing properties at the *ortho* position of the main phenyl, or maintaining the same ring as unsubstituted, is preferred (see **11a**, Figure 4).

On the other hand, the *m*TAAR1 selectivity of compound **11c** (Ar = 2-Cl phenyl; *m*TAAR1 = 2.66 μM) and **11d** (Ar = 2-pyrimidinyl; *m*TAAR1 = 9 μM), bearing an electron-rich moiety as the 2-Cl-phenyl substituent or pyrimidine group, seemed to be achieved through polar contacts involving the T83 sidechain. 

The same research group further elaborated on the biguanide scaffold in light of a novel pharmacophore model developed on a set of potent oxazoline discovered by Roche [54], guiding the design of the novel, and more potent, TAAR1 agonists **12** [44]. Initially, the previously mentioned oxazolines were explored in terms of geometry and conformation energy by means of the systematic Conformational Search tool of the MOE software in order to develop the following pharmacophore analysis. Then, a pharmacophore model was calculated using the pharmacophore search module implemented in the MOE software, starting from the alignment of the aforementioned oxazolines onto the most potent one, taken as reference compound. Based on this information, a set of putative TAAR1 agonists (**12**) were designed. The most interesting analogue developed (**12q**) is reported in Table 2, entry 5.

Briefly, the biguanide scaffold was simplified to the amidino group, while the aryl-piperazine ring was maintained and decorated with several substituents (Figure 4). In vitro tests on *h*TAAR1 revealed the bioactivity of most of them, several of which displayed nanomolar activity (up to 20 nM). The docking of **12q** in the previously mentioned *h*TAAR1 HM [40] was performed to support the results of the in vitro tests: the replacement of the biguanide with an amidino group was shown to be advantageous. Indeed, the most promising derivative **12q** moved the amidine moiety into the proximity of the *h*TAAR1 H99 and D103 residues, detecting H-bond contacts (Figure 4). In addition, the folded piperazine, in tandem with the presence of substituents at the phenyl ring, were projected towards I104, F185, S198, W264, F267, F268, and I290 featuring π–π stacking and Van der Waals contacts.

**Figure 5:**
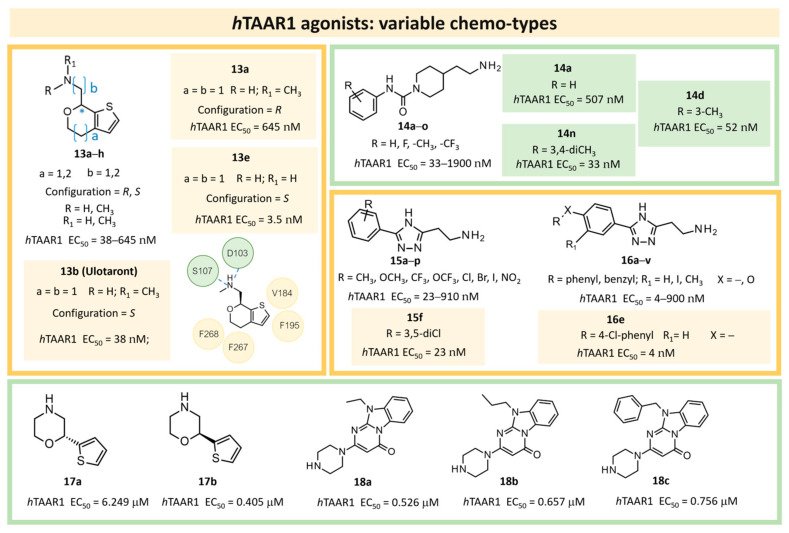
Scheme of the screened TAAR1 ligands **13** exhibiting the main chemo-type of **Ulotaront** (**13b**) [45], of the piperidine-containing compounds **14** [46], and of the triazole-based TAAR1 agonists **15**, **16** [47]. The chemical structure of the recently reported morpholine-based compounds **17** [48] and pyrimidinone-benzimidazole derivatives **18** [50] are shown. Ligplot of **Ulotaront** is depicted, and the most important polar and hydrophobic residues are reported in green and orange, respectively.

More recently (2022), Heffernan et al. reported a retrospective study on **Uloratont** (Figure 5) to investigate its interaction mode with the target and to explore the SAR of this successful chemo-type [45]. 

**Ulotaront** was, in fact, discovered via the in vivo phenotypic approach [22], whereas the molecular target was revealed at a later stage [22]. The reference compound **Ulotaront** was docked in an *h*TAAR1 HM retrieved from the GPCRdb website [55] and built using the β_2_-ADR (PDB ID = 3SN6) [56], with the loops modeled on D_2_ dopamine receptor (D_2_R) (PDB ID = 6CM4) [57], 5-HT_1B_ (PDB ID: 4IAQ) [58], and adenosine A_2A_ receptor (PDB ID: 4UHR) [59]. 

The obtained TAAR1–**Ulotaront** complex was submitted to simulated annealing molecular dynamics (MD), using a hybrid QM/MM model to enhance the accuracy in the binding site description. In particular, the starting pose for **Ulotaront** was determined by docking with the program FRED43 (v4.0) [60] while MD calculations were performed using the AMBER44 (v20) [61] simulation package. Based on the reported studies, the importance of a salt bridge interaction involving D103 was confirmed, as the **Ulotaront** bicyclic core projected towards V184, F195, F267, and F268 (Figure 5). 

Some **Ulotaront** analogs were designed and tested in vitro based on in silico screening (Figure 5). Interestingly, one of the tested compounds exhibited an increased EC_50_ value (up to 3.5 nM) (Table 2, entry 6), compared with **Ulotaront** (38 nM). In terms of SAR analysis, the results pointed out the effective role played by the choice of a primary amine group tethered to the main **Ulotaront** scaffold, as experienced by **13e** (*h*TAAR1 EC_50_ = 3.5 nM; Figure 5). This property should be accompanied by the *S* configuration, rather than to the *R* one. On the contrary, the expansion of the dihydropyran ring of **Ulotaront** to the tetrahydrooxepin ring impaired the ligand potency, compared with racemic **Ulotaront**. Finally, moving the sulfur position in the five-membered ring also proved to be disadvantageous to achieve TAAR1 activation.

In the same year, Krasavin et al. reported the discovery of a series of urea derivatives (**14**; Figure 5) via high throughput screening (HTS) and the subsequent hit expansion of **14o** (Table 2, entry 7) [46]. The SAR of the novel series was investigated by the in vitro testing of further analogs, of which the most potent (**14n**; Figure 5) displayed an EC_50_ of 33 nM. Moreover, a subset of the tested compounds was evaluated in silico to aid SAR rationalization. In the present case, the structural information was obtained by downloading the AlphaFold model for *h*TAAR1 (structure id: Q96RJ0) [62,63]. The protein model thus obtained was preprocessed with the use of the protein preparation wizard, included in the Schrödinger Suite (NY, USA, version 2021-4). The compounds were docked in the predicted structure by means of Glide [64], and, in addition to the docking score, an MM/GBSA procedure for the estimation of the free energy of the binding was applied. Both the examination of the docking poses with respect to a reference compound (**Ralmitaront**) and the free energy calculation allowed for the discrimination between active and inactive compounds. The most promising compounds were also evaluated in vivo, revealing the 3,5-dimethyl-phenyl-substituted analogue (**14o**) as featuring a statistically significant and dose-dependent reduction in hyperlocomotion in DAT-KO rats. 

A similar study was performed by the same group starting with the triazole scaffold featured by **15**, **16** (Figure 5), with **16e** being the most promising individuated from HTS (see Table 2, entry 8) [47]. Compound **16e** exhibited an EC_50_ of 4 nM, being 30-fold more potent than **Ulotaront**. Among compounds **15**, **16**, those bearing the biaryl moiety proved to be more effective than the phenoxy substituted ones. The most promising, **16e**, was investigated in silico, to deepen the knowledge of its interaction mode. Again, the *h*TAAR1 AlphaFold-predicted structure was utilized (ID: Q96RJ0) [62,63]. For all ligands, possible protonation states were calculated with the use of the Epik module of Schrodinger Suite [65]. Ligand docking with the prepared TAAR1 protein model was performed with the use of a Glide induced-fit docking (IFD) method [66].

Compound **16e** was submitted to the IFD docking step, and the resulting complex was submitted to MetaDynamics to verify the persistence of the interaction network hypothesized via IF docking. According to the docking pose, the aromatic-rich structure of the ligand forms several lipophilic contacts with F185, F186 and F195, F267, and F268, as well as a π–π stacking interaction with the F267 aromatic ring. Additionally, a salt bridge is observed with the backbones of D274 and I281, and the sidechain of D274 itself. Compound **16e** was also evaluated in vivo, displaying pronounced effects on the locomotor activity of MK-801-treated Wistar rats. 

Very recently (2023), Wang et al. used the AF model of *h*TAAR1 for a prospective VS campaign [48]. More than one thousand low molecular weight molecules displaying similarity to **Ulotaront** [22] (Tanimoto index > 0.5) were retrieved and docked in the *h*TAAR1 model. 

These calculations were conducted using the LibDock module in Discovery Studio 2018 [67]. The active site in TAAR1 was defined based on the known key residue D103 [68]. All compounds were prepared for docking simulation to consider appropriate protonation states, charges, and energy minimization. Among the top-scored molecules, two candidates (**17a** and **17b**; Figure 5) were selected for MD evaluation (Table 2, entry 9). In silico analysis revealed a favorable interaction pattern for compound **17b**, involving the formation of two H-bonds to D103, as well as favorable π–π interactions between the thiophene moiety and several aromatic residues (F195, F268, and W264). The two compounds were submitted to in vitro analysis, revealing EC_50_ values in the low-μM to sub-μM ranges (**17a** = 6.249 μM, **17b** = 0.405 μM). Moreover, they were evaluated against 5-HT and dopamine D2-like receptors, responsible for important off-target activities of traditional antipsychotic drugs. Compound **17b** exhibited a desirable selectivity profile, and its evaluation was pursued with an in vivo efficacy and pharmacokinetics study.

In the same year, Cichero et al. performed a combined structure-based and ligand-based study to investigate the differences between the *h*TAAR1 and α_2_-ADR in a drug design perspective [49]. Comparative docking calculations of the dual agonist **S18616** [69], as well as of a series of imidazoline/imidazole-based compounds [53,54] with various activities and selectivity profiles, were performed. The X-Ray data of the α_2_-ADR receptor (PDB code = 6KUY) [70] and the AlphaFold model of *h*TAAR1 (AF-Q96RJ0-F1) [71] were exploited.

Molecular docking simulations at the α_2_-ADR receptor were performed by means of the DOCK module implemented in MOE software (2019.01 version), applying the template-based approach. The co-crystallized α_2_-ADR ligand was taken as a reference compound. As regards the *h*TAAR1 AF model, the corresponding binding site was selected based on superimposition to the α_2_-ADR protein, via Blosum62 (MOE software, 2019.01 version) [31].

In addition, the mentioned collection of agonists was utilized to produce two QSAR models, considering the response towards *h*TAAR1 and α_2_-ADR. The two final models were derived applying the chemoinformatic and QSAR packages of MOE. The calculated 302 molecular descriptors were managed using the chemometric package PARVUS [72] for checking the constant predictors, splitting the data into training and test sets, and selecting the most informative molecular descriptors.

According to the obtained data, **Guanfacine** (Table 2, entry 10) was reported as a potent dual TAAR1/α_2_-ADR agonist. Interestingly, the compound bioactivity was maintained in vivo.

In 2024, the same group explored an SAR rationalization of a series of amino-oxazoline TAAR1 agonists produced by Roche [50] by docking in the AlphaFold predicted structure of *h*TAAR1 [62,63]. All the molecular docking simulations at the *h*TAAR1 AF protein model were performed by means of the DOCK tool included in the MOE software [31], via a template-based approach using the previously described **S18616**-TAAR1 complex. According to the observed information, key requirements were determined for the *h*TAAR1 ligands, guiding the discovery of a novel chemo-type for the design of new agonists (see Table 2, entry 11). Consequently, a small set of pyrimidinone-benzimidazoles (**18**, Figure 5) was evaluated via ligand-based methods (FLAP2.2.1. software ligand-based module) [73,74]: molecular interaction fields’ (MIFs) compatibility with the reference compound **S18616** was estimated. In addition, molecular docking calculations of compound **18** in the AlphaFold predicted structure were conducted. The results highlighted the low- to sub-micromolar activity of chemically novel compounds such as **18a**–**c** derivatives. 

In particular, the choice of the piperazine basic ring in the presence of a small hydrophobic chain in N (10) led to the most promising analogues **18a**, **18b** (*h*TAAR1 EC_50_ = 526–657 nM) exhibiting beneficial Van der Waals contacts and π–π stacking with the hTAAR1 binding site. Removing the piperazine ring impaired the potency of the congeners lack of promising TAAR1 agonist ability.

## 3. Structural Information of TAAR1 as Druggable Target

### 3.1. Theoretical Models of TAAR1: An Overview

Several in silico-produced models of TAAR1 were used in drug discovery campaigns: two HMs were built for *h*TAAR1 [40,41], and one for *m*TAAR1 [27].

In addition, the AlphaFold-predicted structure of *h*TAAR1 and one *h*TAAR1 model by GPCRdb [75] were used as well. Most of the models employed a single X-Ray structure to model the target, more frequently choosing an agonist-bound receptor as a protein template. The most utilized is an X-Ray of the human β_2-_ADR, covalently bound to an irreversible agonist, (3PDS) [30] or in complex with Carazolol (PDB ID = 2RH1) [52], or with the high-affinity agonist BI-167107 (PDB ID = 3SN6) [56]. 

In particular, two computational studies [33,41] highlighted the possibility of retrieving both agonists and antagonists by VS on models built with template structures containing exclusively an agonist [33] or a partial inverse agonist [41], applying a combined ligand- and structure-based approach. Accordingly, structurally significant explanations for agonist-bound and antagonist-bound conformations of TAAR1 are thought to be limited. Notably, this information was then supported by crystallographic evidence for aminergic receptors [76] and discussed by Laeremans et al. [77]. Among the reported examples, Costanzi and Vilar [78] carried out a retrospective VS study on the α_2_-ADR, to verify the capability of different conformations of the receptor to enrich the ranking of agonists and antagonists over each other and with respect to decoys. It has been shown that the α_2_-ADR inactive conformations in complex with an inverse agonist (2RH1) or antagonists (3NYA) were able to discriminate antagonists from agonists. The active conformation (3P0G), conversely, was able to enrich agonists over antagonists. The inactive state associated with an irreversibly bound agonist (3PDS), however, did not discriminate between agonists and antagonists. Noticeably, the 3PDS structure was used in most of the HMs built for *h*TAAR1, and the related VS approaches based on this conformation retrieved both agonists and antagonists. The 2RH1 PDB was also used to model TAAR1, and the subsequent VS again retrieved mixed agonists/antagonists, in contrast with the mentioned conformation evaluation. More recently (2019), Scharf et al. [79] reported the possibility of using multiple active conformations of the receptor to favor the discovery of an agonist, always considering the α_2_-ADR.

A perspective of the developed *m/h*TAAR1 theoretical models is reported in Table 3. The percentage of identities with respect to the exploited protein template was calculated by aligning the two sequences with the BLAST-p algorithm [80,81,82]. The sequences were retrieved by the proper Uniprot entries [83]. The BLOSUM62 matrix [84] was used for the alignment, with a gap existence penalty of 11, and a gap extension penalty of 1. The conditional compositional score matrix was used to consider the different amino acid compositions of the query with respect to the frequencies used for the calculation of the substitution matrices [85]. The word-size was set to 3.

Since the Cryogenic Electron Microscopy (Cryo-EM) data of the *h*TAAR1 and *m*TAAR1 were recently solved, it is possible to compare the experimental structures of TAAR1 with the templates utilized for TAAR1 HM building and ligand design. Considering *h*TAAR1, the templates employed for homology modeling mainly included the β_2_-ADR (PDB IDs: 3PDS, 2RH1, 3SN6) [30,52,56]. In addition, the *m/h*TAAR1 AlphaFold modelled structures (AF) were analyzed as well [62,63].

**Figure 6:**
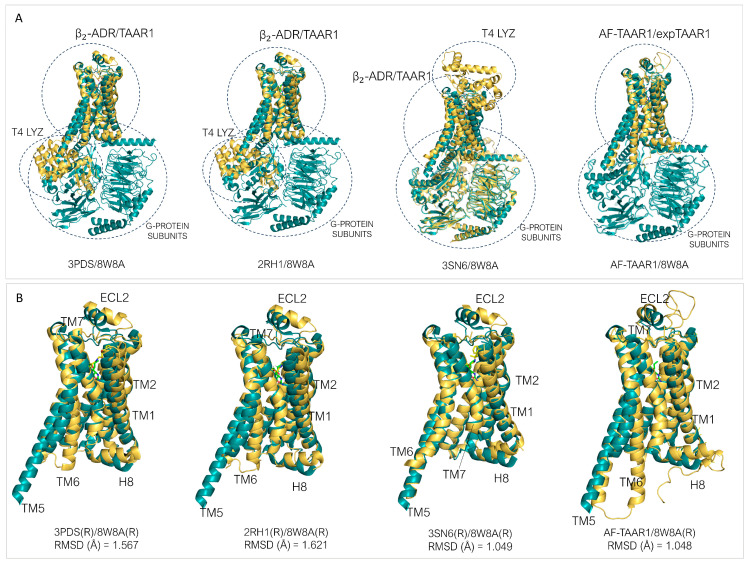
(**A**) Superimposed structures of *h*TAAR1 Cryo-EM structure (8W8A) [87], green) and the templates (yellow) used to generate the *h*TAAR1 HMs (3PDS [30], 2RH1 [52], 3SN6 [56], and the AF model for *h*TAAR1). (**B**) Detail of the superimposition focused on the receptor.

As regards *h*TAAR1, the PDB IDs 3PDS [30] and 2RH1 [52] contain the coordinates of the β_2_-ADR protein- *Tequatrovirus* T4 lysozyme (LYZ), in complex with an irreversible agonist and a partial inverse agonist, respectively (Figure 6A, template in yellow). The 3SN6 PDB [56] also reports the T4 LYZ, but in this case, the receptor is associated to a G-protein (Figure 6A, template in yellow). The 3SN6 structure is in complex with the BI-167107 agonist [56]. The *h*TAAR1 AlphaFold structure only involves the receptor in the apo-form. 

In the Cryo-EM structures of *h*TAAR1, such as in PDB code 8W8A [87], the protein target is associated to the three subunits of a G-protein (Figure 6A, experimental *h*TAAR1 protein in green). By observing Figure 6B, it is possible to highlight an overall agreement in the receptor folding between the experimental structure of the target and the templates as transmembrane domains (TMs), but still, some helices (Hs) and/or extracellular loops (ECLs) significantly deviate from the *h*TAAR1 conformation.

For the 3PDS/TAAR1 systems, ECL2, H8, TM6, TM1, and TM2 exhibit larger discrepancies (Figure 6B). A very similar situation is observed for 2RH1. Conversely, 3SN6 displays a more adherent conformation to TAAR1 with discrepancies concentrated prevalently at ECL2 and H8. The *h*TAAR1 AF structure exhibits the best fit to the experimental TAAR1 structure, exhibiting the lowest α-carbon atom RMSD value among the analyzed templates. TM6, however, deviates importantly from the template structure. Such a helix is considered the hallmark of class A GPCR activation; in particular, its shift outwards is an indication of an activated state, while a straighter positioning is indicative of an inactive state [88,89]. 

**Figure 7:**
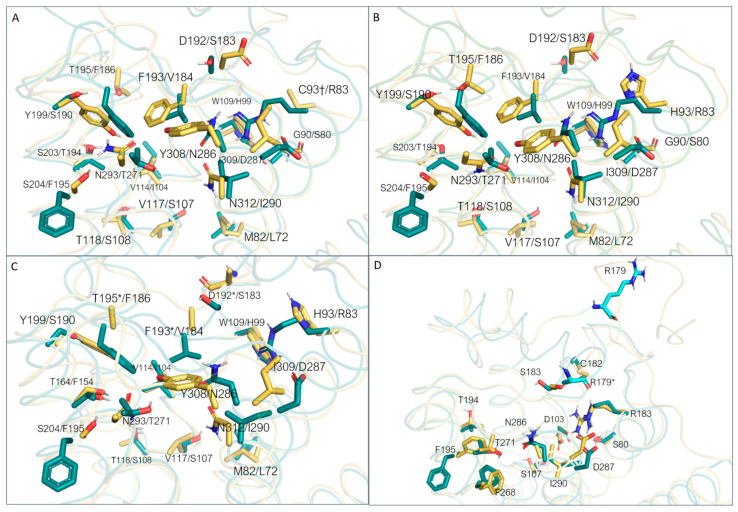
Binding site comparison between the experimentally solved structure of *h*TAAR1 (green, 8W8A) [76] and the templates (yellow) used for HMs, such as (**A**) 3PDS [30], (**B**) 2RH1 [52], (**C**) 3SN6 [56]. (**D**) The AF-predicted structure [62,63] (last update 2022-11-01, id: Q96RJ0, yellow) is compared with 8W8A. Residues of the AF model far from the proper experimental positioning are shown in cyan. Incompletely solved amino acid sidechains were marked with a star symbol. In figures (**A**–**C**), the labels report the amino acid of the template first, and then the amino acid of the *h*TAAR1 Cryo-EM structure.

In the 3PDS and 2RH1 data, the template protein has been reported in the inactive state, and the AF-predicted structure also presents an inactive-like conformation. 3SN6, on the other hand, exhibits the active conformation. The fact that an agonist-bound form, such as 3PDS, can assume the inactive conformation is coherent with experimental data reporting that agonist binding alone is not sufficient for GPCR complete activation, as the intracellular binding to G-proteins or stabilizing peptides is also required for complete receptor activation [30,76]. Regarding the binding site, it is possible to verify that a good correspondence is reached in terms of the superimposition of conserved amino acids. However, some differences can be highlighted as shown in Figure 7. 

For 3PDS, many non-conserved residues are present (Figure 7A). The template/8W8A S203/T194, V114/I104, T118/S108, N312/I290, and W109/H99 couples are shown to exhibit a certain agreement in terms of spatial positioning. For other residues, important differences in terms of steric and/or electrostatic properties, and/or backbone position, arise (Y199/S190, T195/F186, D192/S183, N293/T271, F193/V184, V117/S107). As can be expected, backbone displacements are more easily observed for residues towards the extra-cellular region with respect to more buried residues. In this PDB, residue H93 was mutated to cysteine to anchor the β_2_-ADR irreversible agonist. Apart from this difference, a similar situation can be observed for 2RH1 and 3SN6, as the superimposed protein is the same. In the case of 3SN6, several residues composing the binding site were not completely solved. As the AlphaFold structure of TAAR1 was predicted based on the TAAR1 sequence, this analysis is not extendable to this case. However, although conserved, many amino acids display different orientations with respect to the experimental structure. The most relevant cases are related to residues F195 and R179 (ECL2). 

**Figure 8:**
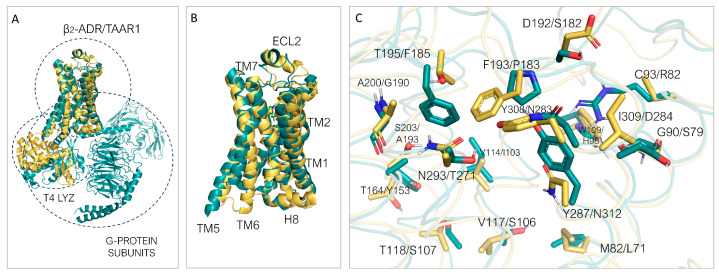
(**A**) Superimposed structures of *m*TAAR1 Cryo-EM structure (green, 8JLK) [90] and the template (yellow, 3PDS) [30], used to generate the previous *m*TAAR1 HMs. (**B**) Detail of the superimposition focused on the receptor. (**C**) Residue comparison at the binding site. The template residues are reported first, followed by TAAR1 corresponding amino acid.

Regarding *m*TAAR1, the utilized template is again β_2_-ADR (PDB ID: 3PDS) [30]. When superimposed to *m*TAAR1, the template shows an overall accordance with respect to the target conformation (Figure 8A). A few discrepancies can be observed between the template and mTAAR1 ECL2, TM1, TM2, TM3, and TM6, with the latter highlighting again the different activation states of the template (inactive) and the *m*TAAR1 (active). 

In terms of residue conservation, the situation is highly superimposable onto the *h*TAAR1, since most of the binding site residues are conserved in the murine orthologue. However, h/mTAAR1 binding sites differ in four amino acids (A193, Y153, P183, and Y287). These residues are also non-conserved in the β_2_-ADR (PDB ID: 3PDS) in which the following substitutions are observed: A193 to S203, Y153 to T164, P183 to F193, and Y287 to N312. Further information on residue conservation between the human and mouse orthologues are reported in Section 3.3.

### 3.2. TAAR1 Experimental Data and Mutagenesis Information

To date, thirteen Cryo-EM structures containing hTAAR1 are available, as well as ten *m*TAAR1 structures (Table 4). The resolution range varies from 3.52 Å (8JLO) [90] to 2.6 Å (8W88) [87]. All the reported structures are associated with an agonist (involving several chemo-types) and with various G-proteins. They all exhibit activated conformation.

**Figure 9:**
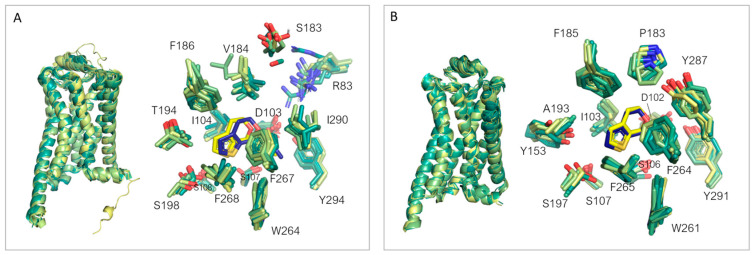
Superimposed structures of the available PDBs of *h*TAAR1 (**A**), and *m*TAAR1 (**B**). High three-dimensional similarity is observed for the overall receptor structure and binding site residues. (PDB IDs *h*TAAR1: 8WCA [91], 8WC8 [91], 8W8A [87], 8W89 [87], 8W88 [87], 8W87 [87], 8JSO [90], 8JLR [90], 8JLQ [90], 8JLP [90], 8JLO [90], 8JLN [90], 8UHB [92]. PDB ID mTAAR1: 8JLJ [90], 8JLK [90], 8WCC [91], 8WCB [91], 8WC9 [91], 8WC7 [91], 8WC6 [91], 8WC5 [91], 8WC4 [91], 8WC3 [91]). The color code follows a gradient from green to yellow. Two conformations of the ligand Ulotaront (yellow, blue) are reported in sticks.

By superimposing all thirteen *h*TAAR1 available structures (Figure 9A), a good agreement of the reported protein conformations can be noticed. As previously reported by Xu [90], the binding site residues themselves display an overall rigidity in response to the binding of different chemo-types. In particular, the deeper portion of the binding cavity is particularly fixed (residues W264, Y294, F267), and other amino acids display small (S107, S108, F186, F268, I290, I104) or important (T194, S189, R83) differences in sidechain conformations but a superimposed position of the backbone. 

Conversely, residues belonging to ECLs (V184, S183) exhibit larger displacement in the backbone positioning. A similar analysis can be extended to *m*TAAR1, (Figure 9B), by comparing the receptor conformations and residue positioning when in complex with different ligands. Again, a close agreement is observed in the overall conformation of the protein. Moreover, the residues composing the binding sites exhibit very similar orientations, except for S197 and a few moderate displacements of Y287, F264, and P183 residues. However, when comparing the binding site rigidity of *h*TAAR1 and *m*TAAR1, it is necessary to consider the larger chemical diversity of the ligands in the case of *h*TAAR1 with respect to mTAAR1, as well as the number of superimposed structures (thirteen vs. ten).

In addition to the abundance of structural information, extensive mutagenesis experiments have been carried out to clarify the role of the binding site residues in *h/m*TAAR1 activation by several chemo-types. For *h*TAAR1 agonism, the flexible phenylethylamine-based derivates, methamphetamine (METH), β-PEA, and amphetamine (*S*-AMPH), have been evaluated, as well as the bulkier and more rigid Ulotaront, RO5256390, and T1AM [87]. The results for *h*TAAR1 are reported in Table 5 [87]. 

The measurements come from different experiments and the percentages are related to different maximum activation values; some data exhibit significant experimental uncertainty (star-labelled in Table 5). However, it is possible to qualitatively compare the importance of the mutated residues with respect to various chemo-types. 

As an example, it is possible to highlight that for all the analyzed ligands, the mutation to alanine of residues D103, I104, S107, W264, and Y294 is detrimental for *h*TAAR1 activation. This turns in key contacts, guaranteed by aromatic and protonable moieties in the *h*TAAR1 ligand, as required features. Residues F186, T194, R83, and H99 also affect the agonist binding, except for *(S)-*AMPH. This could be explained based on the possibility of displaying cation–π contacts or additional H-bonds, thanks to the agonist-protonated nitrogen atom. In the case of *(S)-*AMPH, the basic group is quite hindered by the methyl group if compared with the other agonists.

The I290 mutation to T or N strongly impairs activity for METH, β-PEA, Ulotaront, and RO5256390. In this case, no data are available for T1AM and *(S)-*AMPH. However, the latter is affected by the I290A mutation. The S80A mutation has a strong effect on T1AM, as well as various ranges of activation for the other ligands (no data for *(S)-*AMPH), probably as a key H-bonding feature. The F267A mutation has a strong impact on β-PEA, RO5256390, and T1AM activation, and a lesser (but still significant) impact on METH and Ulotaront. On the contrary, a limited effect is observed for (*S*)-AMPH. 

The F268A mutant was produced to assess only the agonism ability of T1AM and (*S*)-AMPH, leading to a strong and partial reduction in the protein activation, respectively. A similar situation is observed for V184A. Moreover, the mutation of S107 to C was evaluated for METH, β-PEA, SEP-363856, and RO5256390, leading to a strong decrease in activity. This information confirmed the relevant role played by exhibiting H-bonds and π–π stacking between the protein cavity and the ligand, which has to be endowed with limited dimensions and steric hindrance to fit the protein crevice.

Accordingly, S108A mutation was shown to decrease T1AM-induced activation of TAAR1, whereas a partial reduction was observed in response to the F185A mutation on β-PEA, Ulotaront, and RO5256390. Interestingly, the S198A mutation leads to a complex effect for all the ligands excluding *(S)-*AMPH (no data), exhibiting no effect or an increased activation in response to ligand stimulation. Mutational effects regarding METH, β-PEA, Ulotaront, and RO5256390 were evaluated by means of miniGs’ recruitment tests in [87], whereas data regarding T1AM and (*S*)-AMPH were evaluated by CAMYEL assays [90]. In addition to the reported results, mutational data are available regarding the effect of three mutations (S80A, R83A, and H99A) on *h*TAAR1 activation by Fenoldopam, A77636, and Ralmitaront. In all three cases, the mutations appear to have had a moderate to strong influence on ligand-induced activation [90].

Extensive mutagenesis was also performed for a series of key compounds for *m*TAAR1 activation [90,91], referring to the protein agonists T1AM, cyclohexylamine (CHA), trimethylamine (TMA), β-PEA, and Ulotaront. The corresponding results for *m*TAAR1 are reported in Table 6 [90,91]. Data regarding Ulotaront and T1AM were obtained by GloSensor™ assays [90], while the remaining mutational data and also **Ulotaront** were obtained by G protein dissociation assays (BRET) [91]. 

Regarding *m*TAAR1 agonism ability, for each ligand explored in the mutagenesis experiments, the most required key contacts include the conserved D102 residue. Accordingly, very small agonists such as **TMA** or **CHA** are mTAAR1 agonists, featuring a poor potency trend towards the human orthologue [93]. In addition, the W261A mutation deeply affected the ability of all the compounds to activate mTAAR1, as previously observed for the corresponding W264 in *h*TAAR1. It should be noticed that the majority of the *m*TAAR1 agonists herein cited exert their agonist role thanks to aromatic residues, such as F264, F265, and Y287, which are reported as affecting the TAAR1 activation. On the contrary, most of the non-aromatic residues analyzed (S107, P183, and A193) poorly affect the ligand binding. On the contrary, interacting with H-bonding and non-aromatic residues in *h*TAAR1 has been previously reported as key to achieving *h*TAAR1 agonism (see previous Table 5). This, in turn, suggests less planar but folded *h*TAAR1 agonists. This information has been previously proposed in the literature via QSAR studies, which have pointed out the effectiveness of more flexible chemo-types for the design of *h*TAAR1 agonists, while more extended and rigid cores should be preferred for the murine orthologue [43]. In addition, the presence of electron-donor groups is thought to improve *h*TAAR1 binding ability via H-bonds with the aforementioned key residues S107, P183, and A193.

### 3.3. Comparison of the Binding Pockets of hTAAR1 and mTAAR1

The recently published structural information involving both *h/m*TAAR1 sheds light on the species-specificity issue, a critical problem in TAAR1 ligand design [93,94,95,96]. The presence of several structures of *h/m*TAAR1 in complex with the same ligand allows a strict comparison of the binding site, possibly leading to species-specific effect rationalization. The murine orthologue *m*TAAR1 was solved in complex with **Ulotaront** (PDB ID: 8JLK) [90] and **T1AM** (PDB ID: 8JLJ) [90], as well as **β-PEA** (PDB ID: 8WC6) [91] and **ZH8651** (PDB ID: 8WC4) [91]. 

The corresponding complexes with *h*TAAR1 are also available with the following PDB IDs: 8JLO including **Ulotaront** [90], 8JLN in presence of **T1AM** [90], 8W89 [87] or 8WCA [76] including **β-PEA**, 8WC8, and **ZH8651** [91]. 

**Figure 10:**
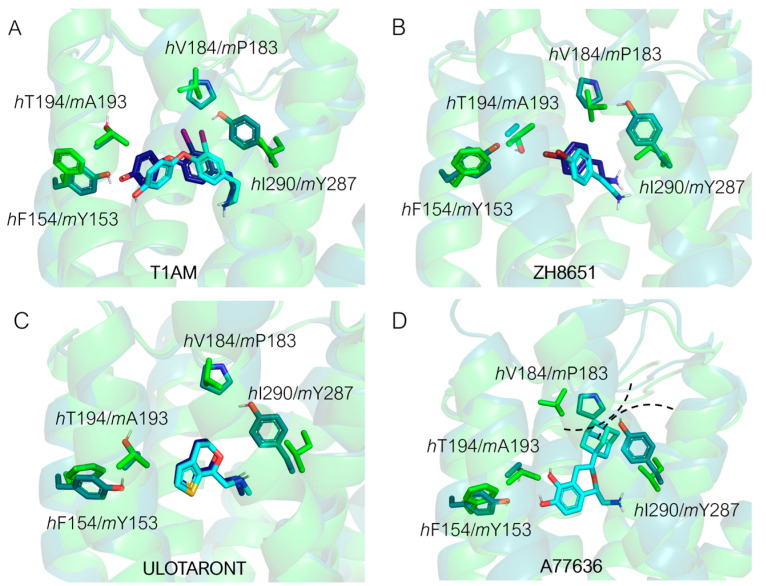
(**A**) Different positioning of **T1AM** in *m*TAAR1 (teal, 8JLJ [90]) and *h*TAAR1 (light green, 8JLN [90]). Residue *h*T194/mA193 was hypothesized to play a role in species-specific affinity differences observed for **T1AM**. (**B**) *h*TAAR1 (PDB ID: 8WC8 [91], light green) and *m*TAAR1 (PDB ID: 8WC4 [91], teal) non-conserved residues around **ZH8651**. (**C**) Superimposition of the human and murine orthologues of TAAR1 in complex with **Ulotaront** (*h*TAAR1: 8JLO [90], green, *m*TAAR1: 8JLK [90], teal). The compound positioning at the *m*TAAR1 and *h*TAAR1 is reported in dark blue and cyan, respectively. The four non-conserved residues are represented in sticks. (**D**) Structural basis of *h/m*TAAR1 selectivity of **A77636** ligand (cyan). The larger hindrance of residues *m*P183 and *m*Y287 (teal, PDB ID: 8JLK [90]) with respect to the corresponding residues in the human orthologues (light green, PDB ID: 8JLR [90]) impairs the positioning of the adamantane substituent of **A77636**, which is in fact inactive towards *m*TAAR1.

The superposition between the three-dimensional structures of the *h/m*TAAR1 in the presence of different chemo-types allows to individuate four non-conserved residues which would help in explaining species-specificity at the protein orthosteric binding site: Y153(*m*)/F154(*h*), P183(*m*)/V184(*h*), A193(*m*)/T194(*h*), and Y287(*m*)/I290(*h*) (Figure 10A–C). 

As shown in Figure 10A, **T1AM**, which exhibits higher potency values towards *m*TAAR1 compared with *h*TAAR1, seems to be better stabilized at the murine orthologue as endowed with more aromatic (Y287) or hydrophobic residues (A193) than *h*TAAR1 (I290 and T194 at the same positions).

Xu et al. provided the structural basis for the selectivity of **A77636**, a catechol derivative reported to be active on *h*TAAR1 and inactive towards *m*TAAR1 [90]. As shown in Figure 10D, the Cryo-EM structure of the *h*TAAR1:**A77636** complex highlights the protruding of the ligand towards residues V184 and I290. These two amino acids are non-conserved in the murine orthologue, being mutated to bulkier residues P183 and Y287, respectively. The larger hindrance introduced in the murine case is reflected in the narrowed shape of the binding pocket in the corresponding area, not allowing the adamantane moiety to be accommodated. These data are further (partially) supported by mutagenesis experiments, as both the *h*V184A and the *h*V184P mutants exhibited impaired **A77636**-induced activation [90]. Such information is critical for the design of novel *h/m*TAAR1 ligands, to control the species-specific aspect. In the same article [90], a rationalization for **T1AM** preference for *m*TAAR1 over *h*TAAR1 was proposed. According to this hypothesis, the non-conserved couple of residues *m*A193/*h*T194 would be responsible for the loss of activity at the *h*TAAR1 with respect to *m*TAAR1. The key role of such residues in species-specificity was previously proposed by computational studies [95,97]. 

**Figure 11:**
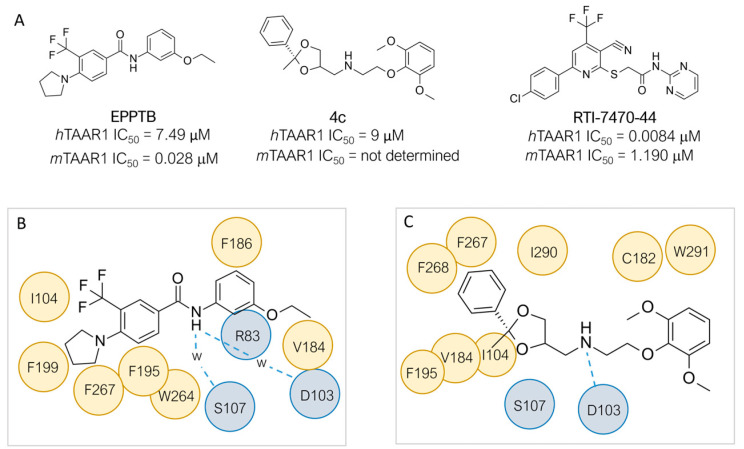
(**A**) Chemical structures of the available *h*TAAR1 agonists: **EPPTB** [98], **RTI-7470-44** [99], and **4c** [33], (**B**) Putative interactions between EPPTB and the orthosteric binding site of *h*TAAR1, investigated by docking and MD [97]. Water molecules mediating H-bonds between the ligand and the receptors were represented as w. H-bonds are represented as blue dashed lines. (**C**) Putative interactions between **4c** and the orthosteric binding site of *h*TAAR1, investigated by docking [33]. H-bonds are represented as blue dashed lines.

In the search for *m/h*TAAR1 antagonists, computational techniques were also utilized to explore species-specificity issues [97]. Indeed, no experimental structures of TAAR1 revealing an antagonist binding mode are available, possibly due to the limited availability of such compounds. Thus, the putative binding modes of **EPPTB** and the antagonist **4c** (Figure 11A), previously reported, were analyzed by docking/MD and docking, respectively [33,97]. The proposed interaction pattern has been reported in Figure 11A–C.

On the other hand, HTS approaches [100] followed by structure-activity optimization allowed for the discovery of the *h*TAAR1 antagonist **RTI-7470-44**, endowed with a species-specificity preference over *m*TAAR1 (Figure 11A) [99]. **RTI-7470-44** displayed good blood–brain barrier permeability, moderate metabolic stability, and a favorable preliminary off-target profile. In addition, **RTI-7470-44** increased the spontaneous firing rate of mouse ventral tegmental area (VTA) dopaminergic neurons and blocked the effects of the known TAAR1 agonist **RO5166017**.

**Figure 12:**
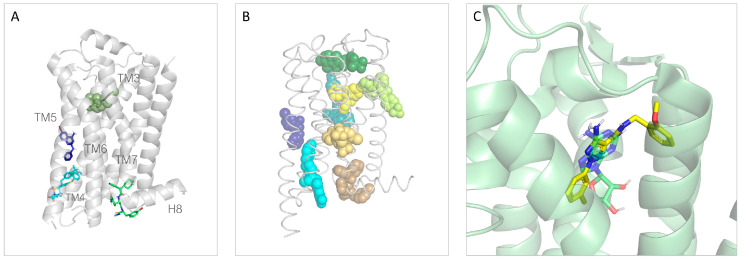
(**A**) β_2_-ADR receptor in complex with the three co-crystallized allosteric ligands **compound-15PA** (green sticks, PDB ID: 5X7D) [103], **compound-6FA** (cyan sticks, PDB ID: 6N48) [104], and **AS408** (blue sticks, PDB ID: 6OBA) [105]. The ribbon structure belongs to the 6OBA PDB ID. The orthosteric binding site is highlighted by the presence of the β_2_-ADR agonist epinephrine, represented in smudge spheres (PDB ID: 7BTS) [106]. (**B**) Non-exhaustive examples of allosteric modulators of class A GPCRs. β_2_-ADR NAM **Compoud-15PA** (brown, PDB ID: 5X7D, [103]), adenosine A1 receptor PAM **MIPS521** (yellow-orange, PDB ID: 7LD3 [107]), CXC chemokine receptor 3 NAM **SCH546738** (cyan, PDB ID: 8HNN [108]), β_2_ADR NAM **AS408** (blue, PDB ID: 8OBA) [105], cannabinoid receptor CB1 PAM **ZCZ011** (yellow, PDB ID: 7FEE) [109], PAR2 allosteric antagonist **AZ3451** (teal, PDB ID:5NDZ) [110], P2Y1R allosteric antagonist **BPTU** (lime, PDB ID: 4XNV) [111], M4 muscarinic acetylcholine receptor PAM **VU0467154** (forest, PDB ID: 7TRQ) [112]. The ribbon belongs to the 8W8S PDB ID [113]. (**C**) An example of bitopic ligand (yellow) with respect to the position of the classical agonist (green). In the present case, the adenosine A_2_a receptor is represented in complex with its endogenous ligand adenosine (PDB ID: 2YDO) [114] and a triazole-carboximidamide bitopic antagonist (PDB ID: 5UIG) [115].

Beyond the design of compounds selectively binding to the TAAR1 orthosteric site, allosteric modulation is gaining attention in the field of GPCR [101,102]. Several allosteric modulators were reported for class A GPCRs, some of them being associated with structural information [101,102]. As an example, three allosteric modulators were co-crystallized in complex with the β_2_-ADR receptor: **compound-15PA** (PDB ID: 5X7D) [103], **compound-6FA** (PDB ID: 6N48) [104], and **AS408** (PDB ID: 6OBA) [105] (Figure 12A). Two of them, the phenylalaninamide **compound-15PA** (Figure 12) and the phenylquinazoline **AS408** (Figure 12), exert a negative allosteric modulation, while the aryl-sulphonamide derivative **compound-6FA** (Figure 12) is a β_2_-ADR positive allosteric modulator.

The identified allosteric site for the β_2_-ADR is formed by TM 1, 2, 6, 7, ICL1, and H8 (cytoplasmatic end of the receptor, 5X7D), by TM 3, 4 and ICL2 (cytoplasmatic end, 6N48), and TM3, 5 (membrane facing surface, 6OBA). However, the known allosteric sites for class A GPCRs exhibit large location variability, taking into account the lipidic interface, the extracellular interface, and the cytoplasmic interface (Figure 12B). 

In addition, ligands that bind both to the orthosteric and protrude towards an allosteric site (bitopic ligands) were reported [102,116]. An example of a bitopic ligand is reported in Figure 12C. A complete discussion of GPCR allosteric sites is beyond the scope of this review, but several valuable reviews are available on the topic [101,102,117,118]. Regarding TAAR1, no allosteric modulators have been reported to our knowledge. However, a putative TAAR1 allosteric binding pocket was hypothesized by Glyakina et al. through a bioinformatic approach [119].

## 4. Computational Methods Guiding the Discovery of TAAR5 Ligands

### 4.1. In Silico Screening of Novel TAAR5 Ligands

Concerning the design of *m*TAAR5 ligands, Cichero et al. reported a VS study [86] of a series of 5-HT_1A_ receptor ligands [34,35,36,37,38,39,120], formerly screened against TAAR1 [33]. Following the same procedure applied for *h*TAAR1 [42], the *h/m*TAAR5 receptors were modelled on the basis of the β_2_-ADR (PDB ID:3PDS), using the BLOSUM62 matrix for the target/template alignment. The structures were minimized, and several parameters were considered for quality evaluation, including the Ramachandran plot analysis, the evaluation of proper distribution of hydrophobic/hydrophilic residues in different areas of the protein, and the rotamer strain energy, among others. The **T1AM** reference compound was docked in the binding site of the obtained model(s) with the use of Sybyl-X1.0, and the best poses were refined by post-docking minimization. Furthermore, the residues around the ligand were submitted to rotamer analysis to explore better conformations. The virtual screening was performed with Sybyl-X1.0. A structural analysis of four HMs (*h/m*TAAR5 and *h/m*TAAR1) was reported to guide the design of isoform-selective and species-specific compounds prior to synthesis. In particular, the binding sites were compared in terms of residues conservation and putative interactions with the reference compound **T1AM**, taking into consideration the different behavior of such compounds towards the considered TAARs. Indeed, **T1AM** has been reported as a TAAR1 agonists, also featuring *h*TAAR5 inverse agonist ability. The screening results were selected according to pharmacophore features based on the previously mentioned structure-based study, and subsequent in vitro tests to individuate two novel *m*TAAR5 antagonists (IC_50_ = 4.8 ± 1.1 μM and 29 ± 1.4 μM). 

The two candidates bear diphenyl-dioxolane (**19**) or tetrahydrofuran (**20**) scaffolds, respectively, and exhibit selectivity with respect to *m*TAAR1 (Table 7, entry 1). 

**Figure 13:**
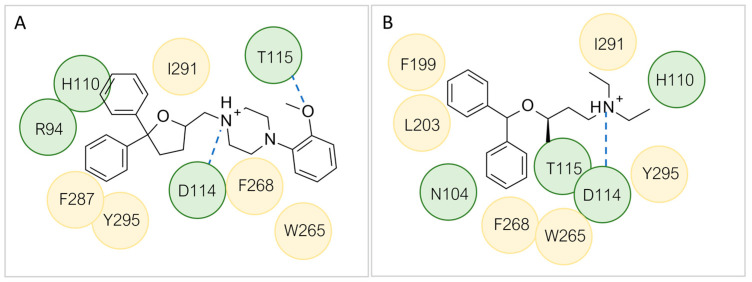
(**A**) Ligplot of the developed *m*TAAR5 antagonist **20** [86] and (**B**) **25** [122]. Polar and hydrophobic residues are reported in green and orange, respectively.

Interestingly, the docking analysis highlighted the presence of specific contacts, such as a H-bond to T115, resulting in the key to TAAR5 selectivity over TAAR1. These data confirmed the key role of T115 in TAAR5, being non-conserved in the *h*TAAR1 orthologue. In addition, the dioxolane and the tetrahydrofuran derivatives **19** and **20** displayed a switched binding mode, maintaining, in any case, a key salt-bridge with D114, through the compound basic moiety. Several π–π stacking contacts with W265, F287, and Y295 were also reported (Figure 13A). 

More recently [121], Bon et al. performed a large-scale VS on a homology model of *m*TAAR5. In detail, several HMs were built using as templates protein structures with sequence identity superior to 32%, and a resolution of at least 3 Å. On the basis of several evaluation parameters such as the alignment coverage, the backbone RMSD with respect to the template structure, and others, the best HM was built on the *Meleagris gallopavo* β_1_-ADR in complex with the agonist **formoterol** (PDB ID: 6IBL) [123]. The screening was carried out with AtomNet^®^ (Atomwise), a structure-based deep convolutional neural network trained for VS purposes, able to predict the affinity of a set of molecules. The binding site was defined on the basis of the ligand position at the template structure. The Enamine In-Stock (https://enamine.net accessed on 23 November 2023) HTS library of around 2 million small molecules was prepared and screened. The top section of the VS results was further submitted to filtering according to various descriptors and clustering with a Tanimoto similarity cutoff of 0.35. Ninety-six compounds were selected among the results, covering different chemical structures. Among the tested compounds, two hits, **21** and **22** (chemical structure not available), were retrieved, exhibiting antagonist behavior [121]. The two compounds experienced IC_50_ = 2.8 μM and 1.1 μM, respectively (Table 7, entry 2). 

In 2023, Nicoli et al. performed a large-scale VS towards a HM of *m*TAAR5 built starting from the crystal structures of the human β_2_-ADR (PDB ID: 4GBR) [124] and the wild turkey β_1_-ADR (PDB ID: 2Y03) [125], exhibiting a structural similarity of 28% and 31%, respectively [122]. 

The ECL2 sequence was modeled based on the ECL2 of neuropeptide receptor Y1 (PDB ID: 5ZBH) [126] (15% sequence identity). MODELLER version 9.25 [127] was employed to generate one hundred possible HMs, and the structure with the best DOPE (discrete optimized protein energy) score was selected. Additionally, the HM was submitted to intra-molecular H-bond optimization at physiological pH with Maestro [128]. The quality of the model was evaluated by various metrics such as Ramachandran plot and steric clashes presence. The model was further optimized through structural and sequence comparison with serotoninergic receptors. Two models (model A and B) were obtained. The SPECS library of approximately 200,000 compounds (https://www.specs.net accessed on 23 November 2023) was prefiltered according to drug-like and pharmacophoric features via Phase by Schrödinger [129]. The filtered compounds were then submitted to docking in the TAAR5 HM(s) with Glide standard protocol [130].

Three compounds (**23**–**25**) (Table 7, entry 3) were found to exhibit antagonistic activity at *m*TAAR5. The corresponding *m*TAAR5 IC_50_ values were 21 ± 0.18 μM, 3.5 ± 0.15 μM, and 2.8 ± 0.16 μM, respectively. Such compounds were further investigated by in-membrane molecular dynamics (MD). For each complex, three replicas of 200 ns in the NVT ensemble were carried out and analyzed.

Compound **23** displayed an ionic interaction with D114 through the charged aliphatic tertiary amine, while the hydroxyl group was H-bonded to D114 too. 

The aromatic moiety was engaged in π−π interactions with F268 and in Van der Waals contacts with L203. The two most potent compounds, **24**, **25**, moved the two aromatic rings towards W265 and F268, as the protonated basic moiety involved in a salt bridge with the key residue D114 (see 25 in Figure 13B).

Based on the above, up to now, three HMs of *m*TAAR5 and one for *h*TAAR5 have been exploited, guiding the search for novel chemo-types acting as TAAR5 ligands. In Table 8, a perspective of the developed HMs in comparison with the selected protein template is reported. 

The percentage of identity was calculated by aligning the two sequences with the BLAST-p algorithm [80,81,82]. The sequences were retrieved using the proper Uniprot entries [83]. The BLOSUM62 matrix was used for the alignment, with a gap existence penalty of 11, and a gap extension penalty of 1. The conditional compositional score matrix was used to consider the different amino acid compositions of the query with respect to the frequencies used for the calculation of the substitution matrices. The word-size was set to 3.

### 4.2. TAAR5: Better Templates for New HMs

To date, no experimental structure have been reported for *m/h*TAAR5. In this case, HMs remain a possible strategy to perform drug discovery campaigns toward the target. In search for novel templates, we performed a sequence search towards *h*TAAR5 with BLAST-P [80,81,82], restricting the query to proteins included in the protein data bank [131]. The results are reported in Table 9, listing the putative template featuring higher similarity to *h*TAAR5 than the most exploited 3PDS code. The results are ordered according to the percentage of identity (% Id) (calculated on 23 November 2023), with each putative template being colored based on the receptor family as follows: TAARs in cyan, β-ADRs in white, α-ADR in orange, and 5-HT receptors in green. The most utilized PDB in the literature to develop TAAR5 models (3PDS) is highlighted in violet and reported in bold. Other alignment metrics are reported, such as the total score (the sum of alignment scores of all segments from the query sequence), the query coverage (a measure of the percentage of the query sequence that has a corresponding residue on the aligned sequence, the closer to 100% the better).

Notably, the recent release of more experimental structures more closely related to TAAR5 opens a promising scenario for the development of improved *h*TAAR5 HMs.

**Figure 14:**
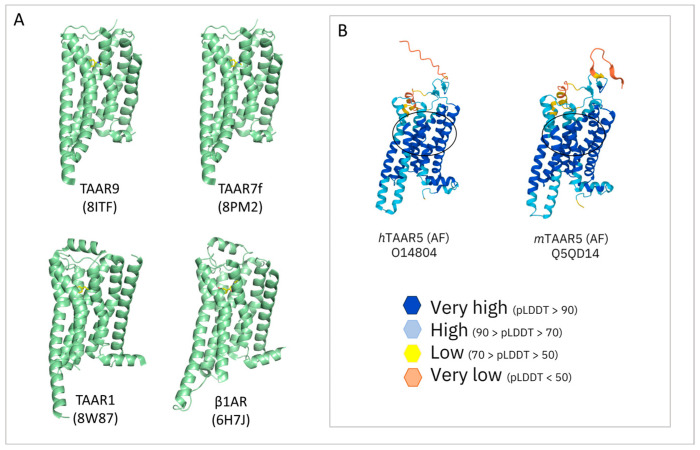
(**A**) Overview of promising GPCR templates for the modeling of *m/h*TAAR5. (**B**) Comparison between the *h*TAAR5 and *m*TAAR5 structures as predicted by AlphaFold. The color code represents the degree of reliability of the prediction (blue: good, orange: bad). AlphaFold per-residue model confidence score (pLDDT) varies between 0 (no confidence) and 100 (very high confidence). As it is possible to notice, an area of low reliability in the prediction can be observed in the area of the orthosteric binding sites of the two orthologues, reported as a black circle.

As expected, the top section of the alignment results is occupied by the available TAARs PDB structures (*m*TAAR9, *m*TAAR7f, *h*TAAR1, and *m*TAAR1). In all cases, the coverage of the sequence is over 95%. 8ITF represents the most promising template for producing an *h*TAAR5 HM according to the percentage identity value (46.45%). However, the sub-optimal resolution of such Cryo-EM structures must be carefully evaluated (3.46 Å). A possible alternative is the second scored result (8PM2, resolution of 2.92 Å). After the TAARs, the β_1_-ADR from various organisms and in combination with various fusion proteins is reported as a promising template, with percentages of identities around 36–37%, and a query coverage of more than 80%. Immediately after, the α-_2A_ ADR and the 5-HT_4_R are proposed, with %ids of 36.14% and 35.45%, respectively. Below this value, several isoforms of the mentioned receptors are proposed, namely the β_2_-ADR, the α_1A_-ADR, and 5-HT_6_R, and the 5HT_2A_R. At a %Id of 33.16%, it is possible to find the previously utilized 3PDS PDB. A few examples of promising templates for the modeling of *h*TAAR5 are represented in Figure 14A. 

Concerning *m*TAAR5, an identical search was performed to individuate novel putative templates for ameliorated homology modelling approaches (Table 10). 

Again, the first positions are occupied by the TAARs (*m*TAAR9, *m*TAAR7f, *m*TAAR1, and *hTAAR1*). Following the TAARs, the β_1_-ADR from *meleagris gallopavo* is proposed. Noticeably, such a template was already utilized by Bon et al. [121] to build a *m*TAAR5 HM, with the last reported PDB ID (6IBL) [123]. The 5HT_2A_R is also considered, with a percentage of identity of 36.70%. Given the much higher similarity and query coverage of the TAARs templates, such molecules can be considered as the best templates for future *m*TAAR5 HMs. Once more, the resolution must be taken into consideration. 

The use of AlphaFold may also be considered for both the two *m/h*TAAR5 proteins (ID: Q5QD14 and O14804, respectively) [62,63]. However, in the previously cited VS campaign [121] directed towards the discovery of *m*TAAR5 antagonists, this possibility was excluded, as the AlphaFold-predicted target structure reported a region with high uncertainty in the proximity of the binding site. Through a comparison of the mentioned *m*TAAR5-predicted structure with its corresponding human orthologue (Figure 14B), it is possible to assert that this aspect may also be relevant for *h*TAAR5, further supporting the development of HMs for the human orthologue.

## 5. TAAR1/5 and Other GPCRs: A Repositioning Perspective

### 5.1. Comparison of hTAAR1 and Druggable GPCRs

In the last few years, several studies have highlighted the potential of repositioning as a valuable strategy for drug discovery [132]. A few attempts involving TAAR1 were also proposed, such as the previously cited 5HT_1A_R and/or α_1_-ADR ligands repositioning [33]. Here, we propose and compare some possible druggable targets to develop the repurposing strategy for the search of novel *h*TAAR1 ligands. This comparison has been conducted based on the percentage of identity (% Id) between the putative template and the *h*TAAR1 protein, choosing % id. values > 30% as the cut-off for the analysis. The search has been performed referring to proteins whose structural information is experimentally available (Table 11). Bearing in mind the previously attempted repurposing methods [54,133,134], we selected the reference proteins shown in Table 11 (in green) in detail: the 5-HT_2A_R (PDB ID: 7WC4, % id: 44.58%, query coverage: 23%) [135], the D(1A) dopamine receptor (PDB ID: 7CKY, %id: 33.55%, query coverage: 82%) [136], the α_1B_-ADR (PDB ID: 7B6W, %id: 32.78%, query coverage: 87%) [137], the β_2_-ADR (PDB ID: 4GBR, %id: 32.65%, query coverage: 85%) [124], the α_1A_-ADR (PDB ID: 7YM8, %id: 31.58%, query coverage: 78%) [138], the Histamine H_2_R (PDB ID: 7UL3, %id: 31.05%, query coverage: 89%) [139], the 5-HT_6_R (PDB ID: 7YS6, %id: 30.58%, query coverage: 85%) [140], and the Muscarinic acetylcholine receptor M3R (PDB ID: 8E9W, %id: 30.57% query coverage: 86%) [141]. 

**Figure 15:**
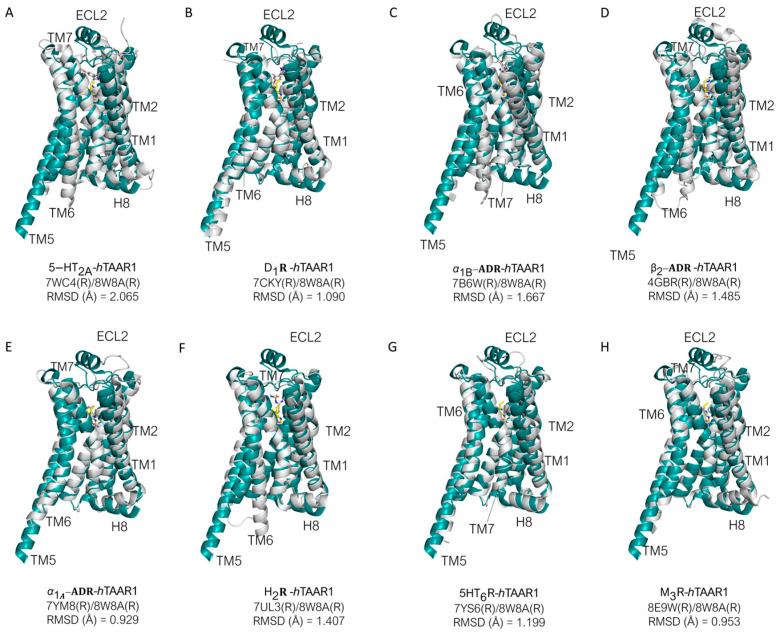
Superimposed structures of *h*TAAR1 (green) and the proposed candidates to repositioning (light gray). (**A**) 5-HT_2A_ and *h*TAAR1, (**B**) D_1_R and *h*TAAR1, (**C**) α_1B_-ADR and *h*TAAR1, (**D**) β_2_-ADR and *h*TAAR1, (**E**) α_1A_-ADR and *h*TAAR1, (**F**) H_2_R and *h*TAAR1, (**G**) 5HT_6_R and *h*TAAR1, (**H**) M_3_R and *h*TAAR1 are compared. The PDB IDs are reported below the names of the superimposed receptors, as well as the Cα RMSD calculated with respect to *h*TAAR1.

The mentioned PDBs were superimposed to the *h*TAAR1 structure (PDB ID: 8W8A) [87]: the results are presented in Figure 15A–H and point out a comparable overall fold of the two receptors. A larger variability is observed for the extracellular region and H8 (except for 5HT6). 

Regarding the TM helices, TM1 exhibits a certain shift with respect to the *h*TAAR1 structure, especially for the adrenergic, 5-HT_6_R, and M_3_R. Minor shifts were observed for TM7 and TM2. Among the analyzed receptors, the dopamine receptor D1 (DRD1), the α_1A_-adrenergic one (α_1A_-ADR), and the muscarinic M_3_R exhibit the lowest Cα RMSD with respect to *h*TAAR1. On the contrary, 5-HT_2A_R represents the highest Cα RMSD structure.

Apart from the overall conformation similarity, the residue conservation at the orthosteric pocket was investigated. The *h*TAAR1 binding site was defined at 5 Å from the reported ligand (PDB ID: 8W8A), including residues T100, I104, V184, F186, D103, R83, V76, M77, S297, L72, Y294, W291, G293, I290, W264, F267, S107, S108, T271, F268, S198, and T194. These residues were compared with the corresponding amino acids at the candidate protein. In addition, residues H99, S80, and S190 were also considered, as they were indicated as key residues for the TAAR binding site [90]. The obtained (non-)conserved residues between *h*TAAR1 and the proposed reference protein are listed in Table 12.

**Figure 16:**
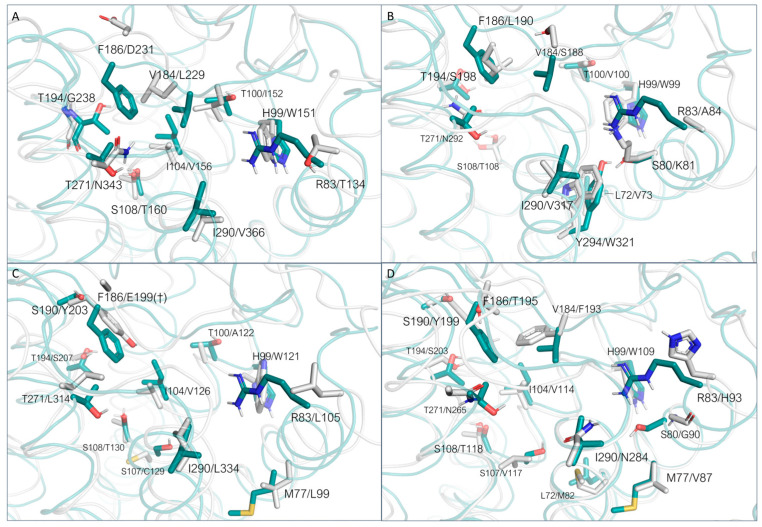
Binding site comparison between *h*TAAR1 (green) and the proposed receptors for repositioning studies (light gray). Only the non-conserved residues are reported. The name of the *h*TAAR1 residue is reported first, followed by the name of the corresponding residue on the analyzed receptor. (**A**) Comparison between *h*TAAR1 and the 5-HT_2A_R. (**B**) Comparison between *h*TAAR1 and the D_1_R. (**C**) Comparison between *h*TAAR1 and the α_1B_-ADR. (**D**) Comparison between *h*TAAR1 and the β_2_-ADR.

The analysis of the 5-HT_2A_R receptor highlighted a rather good conservation rate in the binding site, with few non-conserved residues, such as F186/D231, V184/L229, T100/I152, H99/W151, R83/T134, I290/V366, S108/T160, T271/N343, T194/G238, and I104/V156 (Figure 16A).

However, most of these structural variations maintain comparable polarity and steric properties with respect to the *h*TAAR1 residues. On the other hand, several sidechains of conserved residues significantly differ in their conformations, especially the two phenylalanine residues (F199,TAAR1/F243,5HT_2A_R, F268,TAAR1/F340,5HT_2A_R). A similar situation can be observed for the D_1_R (Figure 16B): T271 is replaced by a N residue, S108 to a T, I290 to a valine, and H99 to a W. Other substitutions are F186/L190, L72/V73, R83/A84, T100/V100, T194/S198, Y294/W321, and S80/K81. Intriguingly, K81′s introduction in place of S81 re-introduces a positive charge in the area previously occupied by R83. In the case of α_1B_-ADR, we can observe the same substitution pattern of residues S108(T), H99(W), and T194(S) (Figure 16C) previously mentioned for 5HT_2A_R and D_1_R receptors. Other non-conserved residues include the *h*TAAR1 R83 which is substituted with a leucine residue and the following ones: T100/A122, I104/V126, F186/E199, T271/L314, and I290/L334. Additionally, M77 is mutated to L99, S107 to C129, and S190 to Y203, introducing a phenyl moiety partially invading the binding pocket. The β_2_-ADR exhibits a larger number of differences in the binding site residues if compared with *h*TAAR1, such as S107/V117, M77/V87, S80/G90, R83/H93, T271/N266, V184/F193, G186/T195, L72/M82, and I290/N284 (Figure 16D). While most of the polarity properties are maintained, the corresponding residue dimensions increase in the β_2_-ADR with respect to the related *h*TAAR1 amino acid. Further mutated residues such as S190(Y), T194(S), S108(T), and I104(V) are in accordance with the reported residues featured by the other reference GPCRs, D_1_R and α_1B_-ADR. 

**Figure 17:**
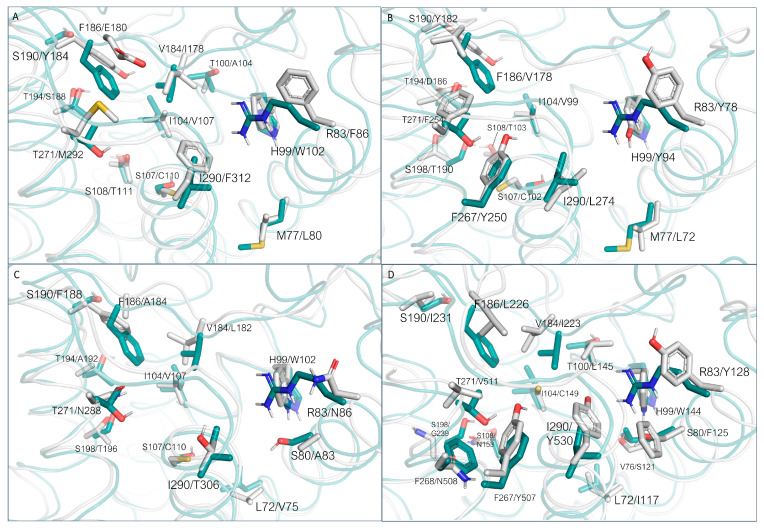
Binding site comparison between *h*TAAR1 (green) and the proposed receptors for repositioning studies (light gray). Only the non-conserved residues are reported. The name of the *h*TAAR1 residue is reported first, followed by the name of the corresponding residue on the analyzed receptor. (**A**) Comparison between *h*TAAR1 and the α_1A_-ADR. (**B**) Comparison between *h*TAAR1 and the H_2_R receptor. (**C**) Comparison between *h*TAAR1 and the 5-HT_6_R. (**D**) Comparison between *h*TAAR1 and the M_3_R.

In the case of α_1A_-ADR, it is possible to observe a large number of substitutions, often coherent with the previously analyzed cases (S190/Y184, T100/A104, M77/L80, S108/T111, H99/W102, I104/V107, T194/S188) (Figure 17A). 

Differences can be observed for residues R83 (substituted with a F residue), I290 (changed to F), S107(C), V184(I), T271(M), and F186(E). The HRH2 receptor exhibits a lower number of previously observed substitutions (S190/Y182, M77/L72, S107/C102, I104/V99, S108/T103), whereas several novel residues are introduced (Figure 17B). H99, in this case, is replaced by a Y residue (Y94), I290 is replaced by L274, T194 by D186, and T271 by F254. R83 is again substituted by an aromatic residue (Y78). In addition, S198 is replaced by a threonine residue. 

The 5-HT6R exhibits a higher similarity of the binding site to *h*TAAR1 with respect to β_2_-ADR, α_1A_-ADR, and H_2_R (Figure 17C). Several residues are substituted with similar amino acids (E.g., I104/V107, S107/C110, S198/T196, V184/L182). Again, it is possible to observe the H99/W102 replacement, as well as the aromatic substitution of S190 (to F188). Other replacements are S80/A83, R83/N86, F186/A184, T194/A192, T271/N288, I290/T306, and L72/V75. The M_3_R exhibits poor residue conservation at the orthosteric site, with respect to *h*TAAR1. Apart from previously observed substitutions (R83(Y), F267(Y), H99(W), F186/L, and V184(I), newly introduced replacements are highlighted (S190/I232, T100/L145, I104/C149, I290/Y530, F268/N508, S198/G239, L72/I117, S80/F125, T271/V511, S108/N153, F267/Y507). Consequently, this receptor would be difficult to target in a repositioning perspective, whereas 5HT_2A_R, D1R, α_1B_-ADR, and 5-HT_6_R exhibit a higher potential in this regard.

### 5.2. Comparison of hTAAR5 and Druggable GPCRs

**Figure 18:**
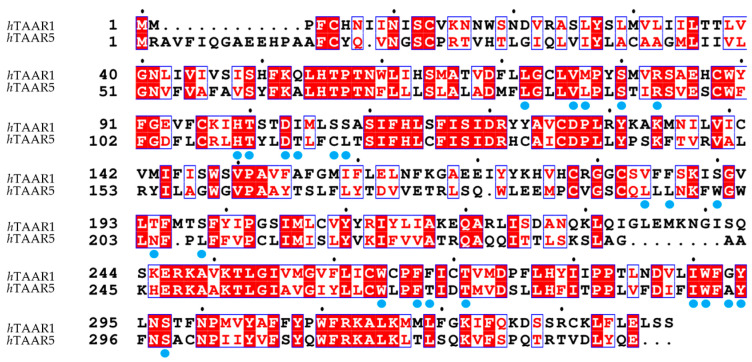
Sequence alignment between *h*TAAR1 and *h*TAAR5. The putative binding site residues of TAAR5 are highlighted with blue dots. The alignment was performed with T-Coffee using the PSI-TM algorithm, with the slow/accurate option. The alignment was performed with the default parameters (BLOSUM62 matrix for the alignment, gap penalty for the creation of a gap of 50 units, no penalty for the gap extension).

Regarding TAAR5, the lack of structural information does not allow for the identification of the protein binding site and to perform a three-dimensional comparison with putative reference GPCRs. However, it is possible to perform a sequence alignment between *h*TAAR1 and *h*TAAR5, to individuate the putative key residues of the *h*TAAR5 binding site. Sequence alignment was performed with T-Coffee [142,143], using the PSI-BLAST algorithm [144,145]. As shown in Figure 18, the *h*TAAR5 binding site may be constituted by residues L83, V87, L88, S91, R94, H110, T111, D114, T115, C118, L119, L194, L196, W200, N204, L207, W265, F268, T269, T272, I291, W292, A294, Y295, and S298.

Out of the twenty-five considered residues, the majority are conserved with respect to *h*TAAR1. Only residues M77(L), I104(T), S107(C), S108(L), V184(L), F186(L), S190(W), T194(N), S198(L), F268(T), and G293(A) are changed. The reported analysis is coherent with the one reported by Xu [90]. The individuated residues also constitute the binding site in the AlphaFold-modelled *h*TAAR5 (ID: O14804) [62,63].

Based on the above, it is possible to compare the proposed *h*TAAR5 binding residues with those of reference GPCRs, paving the way for future drug repositioning strategies. In this case, we performed a search for the most closely related receptors taking *h*TAAR5 sequence as a reference. The search was performed considering sequences associated with a PDB entry, to allow structure-based drug design. The alignment was performed with T-Coffee PSI-TM algorithm, with the slow/accurate option. The obtained (non-)conserved residues between *h*TAAR5 and the proposed reference protein are listed in Table 13. 

Among the results, we selected eight *h*GPCRs of interest (Table 14, green), as these have been extensively studied as druggable targets in medicinal chemistry [146,147,148,149,150,151,152,153], exhibiting identity percentage values (% Id.) > 30% with respect to *h*TAAR5: the α_2A_-ADR (% Id: 36.14%), the β_2_-ADR (% Id: 34.27%), the 5-HT_6_ receptor (% Id: 34.12%), the H_2_ receptor (% Id: 31.58%), the α_1A_-ADR (% Id: 31.27%), the 5-HT_1B_ (% Id: 30.33%), the 5-HT_1A_ (% Id: 30.00%), and the D(1A) dopamine receptor (% Id: 29.09%).

**Figure 19:**
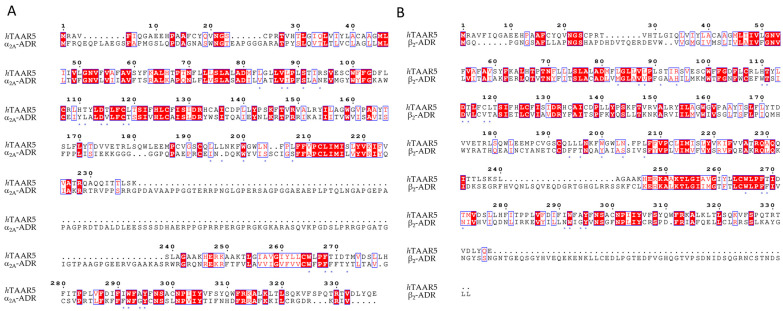
Sequence alignment of *h*TAAR5 and α_2A_-ADR (**A**) and β_2_-ADR (**B**). The putative binding-site residues are highlighted with a star symbol. The alignment was performed with T-Coffee using the PSI-TM algorithm, with the slow/accurate option. The alignment was performed with the default parameters (BLOSUM62 matrix for the alignment, gap penalty for the creation of a gap of 50 units, no penalty for the gap extension).

**Figure 20:**
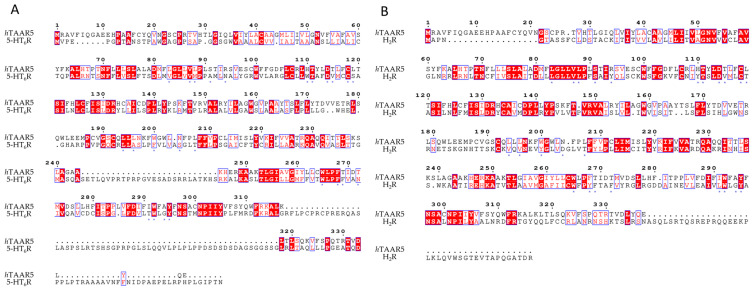
Sequence alignment of *h*TAAR5 and 5-HT_6_R (**A**) and H_2_R (**B**). The putative binding-site residues are highlighted with a star symbol. The alignment was performed with T-Coffee using the PSI-TM algorithm, with the slow/accurate option. The alignment was performed with the default parameters (BLOSUM62 matrix for the alignment, gap penalty for the creation of a gap of 50 units, no penalty for the gap extension).

All the entries presented an acceptable query coverage (over 75%). The sequence alignment of the best ranked couple of *h*TAAR5 and reference GPCRs are reported in Figure 19 and Figure 20 [154]. One kind of adrenergic and serotoninergic subfamily of receptors have been reported, as representative of the corresponding GPCR family.

As shown in Figure 19A, by analyzing the α_2A_-ADR, it is possible to notice a good conservation rate with respect to the *h*TAAR5 binding site.

Residues V87, S91, D114, C118, W200, W265, F268, W292, Y295, and S298 are conserved between the candidate protein and TAAR5. Some other residues exhibit substitution with the same type of amino acids: L83, L88, L194, and A294 are substituted with other hydrophobic residues, H110 with another aromatic residue, and N204 to the polar amino acid serine. Other replacements introduce larger differences, such as hindrance (T269F, T272Y, I291F), different polarity (T111 to L, T115 to V, L119 to T, L207 to S), or protonation state (R94 to N). The sequence alignment did not assign a specific residue corresponding to L196 due to the introduction of a gap. 

The case of β_2_-ADR is almost like the α_2A_-ADR one (Figure 19B), with several residues that are still conserved (V87, T111, D114, W265, F268, W292, Y295, and S298). Among the non-conserved amino acids, aromatic residues are changed to other aromatics (H110 to W, W200 to Y). Concerning the hydrophobic residues, they are substituted with amino acids of the same type (L83 to M, L88 to V, A294 to G) or with polar amino acids (L119 to T, L196 to T, L207 to S, I291 to N). Conversely, some polar amino acids are changed to hydrophobic (T115 to V, S91 to G, C118 to V), whereas some others retain the polar feature (N204/S, T272/N). The basic R84 is changed to one H residue. Finally, a significant hindrance is introduced as the L194 is changed to F, and in the case of T269, again replaced with a F residue.

Concerning the 5-HT_6_R, residues V87, T111, D114, C118, L194, W265, F268, W292, Y295, and S298 are predicted as conserved (Figure 20A).

Again, aromatic residues are substituted with other aromatics (H110 to W, W200 to F). The hydrophobic features of residues L83, L88, L196, and A294 are retained in the 5-HT_6_R (they were substituted by V, M, A, and G, respectively). On the contrary, L119, L207, and I291 are replaced by polar residues (S, T, and T). N204 and T272 are replaced with other polar residues (S and N), while S91 and T115 are changed to non-polar residues (A and V, respectively). The basic R94 is replaced by an N residue. T269 is instead replaced by an F residue, introducing aromaticity and hydrophobicity. Several residues of the H_2_R binding site are predicted to be conserved with respect to *h*TAAR5 (L83, V87, L88, S91, T111, D114, C118, W265, W292, Y295, S298) (Figure 20B). Moreover, most of the introduced changes are more or less compatible with the reference residues of TAAR5. The aromatic residues H110, W200, and F268 are substituted by other aromatic residues (W, Y, and Y, respectively). Many hydrophobic residues are replaced with other hydrophobic amino acid of comparable dimension (L194 to V, L196 to V, I291 to L, and A 294 to G). A shift from hydrophobic to polar is observed for L207(T), and L119(T). Conversely, polar residues T269 and T272 are both changed to a F, and T115 to a V. Polarity is instead retained in the cases of N204(D), while the basic R94 is substituted with a Y residue. Although it is an aromatic residue, the Y still maintains the hydrogen-bond donor moiety due to its R residue, representing an advantage for repurposing. 

In the case of the α_1A_-ADR, the conserved residues are L83, V87, L88, S91, D114, C118, W265, F268, W292, Y295, and S298. The aromaticity is preserved for residues H110 (W) and W200 (Y), and it was introduced at the T269 (F), I291 (F), and R94 (F). Regarding the hydrophobic residues, they are substituted by residues of the same type in the cases of L194(I), and A294(G), while they are substituted with polar residues in the cases of L119(T), L196(E), and L207(S). Conversely, polar residues T111(A), T115(V), N204(A), and T272(M) are changed to non-polar amino acids.

Regarding the 5-HT_1B_R, the number of conserved residues in the binding site decreases to nine (V87, S91, D114, C118, W265, F268, W292, Y295, and S298). Among the non-conserved residues, five hydrophobic amino acids are maintained as hydrophobic (L83 to V, L88 to M, L194 to I, L207 to A, A294 to G), whereas two of them are converted to polar residues (L119 and I291, both converted to T). Aromatic residue H110 is replaced by a tryptophan. Aromatic residues are introduced in place of R94 (changed to Y, again retaining the HBD feature), L196(Y), and T269 (F). Polar residues T111 and T115 are changed to non-polar residues L and I, while polarity is maintained in the case of residues N204(T) and T272(S). A gap is introduced in place of residue W200. In the case of 5-HT_1A_R, the situation is very similar. A few differences can, however, be observed, such as the conservation of residue L88. On the contrary, the previously conserved residue S91 is now non-conserved (changed to A). H110 is again changed to another aromatic residue (F), while W200 is changed to Y. Polar residues T111 and T115 are again substituted by other hydrophobic residues (I and V, respectively), whereas T272 is changed to an A. Finally, non-polar residues L196 and I291 are changed to K, and N, respectively. All the other residues are the same as in the previous case. The D_1_R exhibits a very low conservation rate in the binding site residues, as only 7/25 are conserved (V87, D114, L196, W265, F268, W292, S298), according to our analysis. The majority of the replacements had already been observed for other receptors among the considered ones (L83 to V, L88 to M, H110 to W, T115 to I, L119 to T, W200 to Y, N204 to S, L 207 to S, T269 to F, T272 to N, A294 to G). In the cases of R94 (A), T111(V), C118(S), L194(S), I291 (V), and Y295 (W), new residues were introduced.

In summary, receptors H_2_R and α_1A_-ADR exhibit the highest rate of conserved residues within the binding site with respect to *h*TAAR5 (11/24). However, H_2_R might represent a better starting point than α_1A_-ADR, as the type of introduced residues exhibits a closer resemblance to the original residues found at TAAR5. On the contrary, in α_1A_-ADR, more polarity variations are present. Moreover, the substitution of R94 with a Y residue (H_2_R) might be more compatible with the original R residue with respect to the F substitution (α_1A_-ADR). In addition to these two GPCRs, α_2A_-ADR, 5-HT_6_R and 5-HT_1_R can also be considered. The conservation rate is in these cases of 10/25, 10/25, and 9/24, respectively. The worst predicted match is between the target and D_1_R, which have a conservation rate of only 7/24. Residues V87, D114, W265, and W292 are conserved in all the considered receptors. Y295 is conserved in any of the analyzed cases excluding D_1_R. Conversely, some residues are always mutated (R94, H110, always mutated to other aromatics, T115, L119, L194, L196, N204, L207, T269, consistently mutated to a F, T272, I291, A294, always mutated to G). 

## 6. Conclusions

The present review collects and analyzes the applications of computer-aided drug design tools leading to the discovery of novel ligands targeting *h/m*TAAR1 and/or *h/m*TAAR5. Additionally, when available, the SARs of the discovered compounds are reported. Most of the studies were performed trying to predict the three-dimensional structure of the target by means of homology modeling techniques. Alternatively, the AlphaFold-predicted structures were also utilized. In most of the cases, the target was modeled on the inactive conformation of template GPCRs, such as the β_2_-ADR, or referring to AlphaFold models which were in an inactive-like conformation. 

As the *h/m*TAAR1 experimental structures were solved, we were able to compare the structural information utilized for TAAR1 modeling with the novel structural data. In addition to this retrospective study, we also reported some information of interest for SBDD towards *h/m*TAAR1, comparing the novel *m/h*TAAR1 Cryo-EM structures and the related mutagenesis data, and reporting a possible elucidation of the species-specificity issue from a structural perspective [90]. 

To date, no structure has been solved for TAAR5 and no structural comparison with the previously exploited homology modeling templates has been possible. To improve the quality of HMs of this target, we proposed novel protein templates according to the coupling of protein identity. Along with this, the choice of the TAAR5 AlphaFold alternative has been reported. We have also proposed possible reference targets to guide future drug repurposing for both *h*TAAR1 and *h*TAAR5. Regarding *h*TAAR1, the binding site conservation was discussed based on the structure superposition of the *h*TAAR1 experimental data and the three-dimensional structure of the selected templates. With experimental information on *h*TAAR5 still lacking, protein sequence alignment approaches have been applied, referring to proper template GPCRs. The results are expected to give new hints for the future design of novel, more effective, TAAR1/5 ligands.

## Figures and Tables

**Table 1:** Drug discovery studies focused on *m*TAAR1 agonists involving modelling techniques. The corresponding references (Ref.) are shown; *h*TAAR1 agonism ability was not determined.

Entry	Year	Method of Discovery	Use of the Derived Computational-Based Structural Information	Proposed Hit(s)	mTAAR1 EC_50_	Ref.
1	2015	Rational design (synthesis)	SAR rationalization	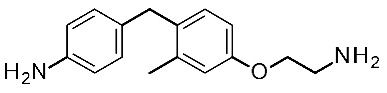 **1c**	240 nM	[27]
2	2016	Rational design (synthesis) combined with previously reported docking analysis	Hit-to-lead optimization	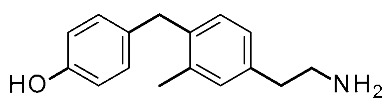 **2b**	98 nM	[28]

**Table 2:** Drug discovery studies focused on *h*TAAR1 involving modeling techniques. The related references are reported (Ref.).

Entry	Year	Method of Discovery	Use of Structural Information	HIT Compound Example	*h*TAAR1 EC_50_ *(IC_50_)*	Ref.
1	2014	VS on *h*TAAR1 HM	Prospective drug discovery, SAR rationalization	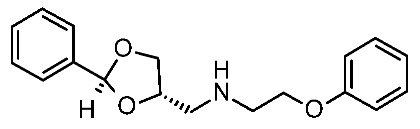 **4a**	2.4 μM	[33]
				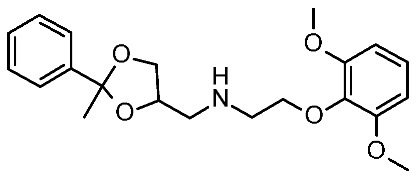 **4c**	*9 μM*	[33]
2	2015	VS on *h*TAAR1 HM	Prospective drug discovery	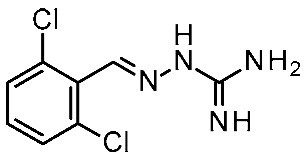 **8b**	0.09 μM	[41]
3	2017	In silico aided-drug design	Prospective drug design, SAR rationalization, selectivity/specificity rationalization	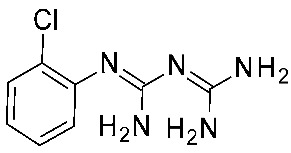 **9a**	1 μM	[42]
4	2018	In silico-aided drug design (QSAR, docking)	QSAR: prospective drug design, docking: selectivity rationalization	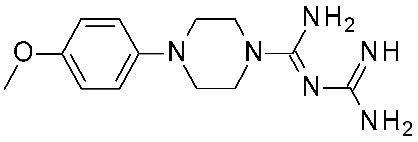 **11h**	11.4 μM	[43]
5	2020	In silico aided drug design (pharmacophore model, docking)	Prospective drug design (pharmacophore model), SAR rationalization (docking)	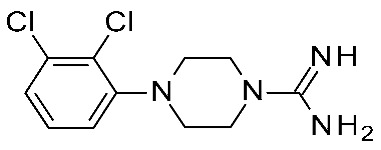 **12q**	20 nM	[44]
6	2022	Hit expansion (synthesis of Ulotaront analogs) *	Study of the mechanism of action of Ulotaront, SAR rationalization.	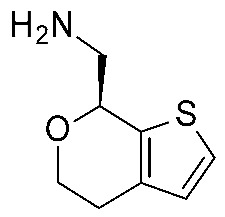 **13e**	3.5 nM	[45]
7	2022	HTS+hit expansion	SAR rationalization	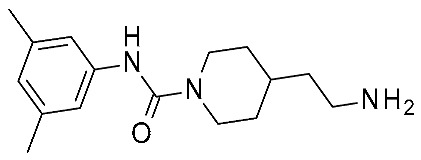 **14o**	112 nM	[46]
8	2022	HTS+hit expansion	Interaction mode investigation	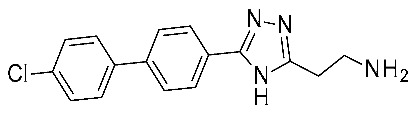 **16e**	4 nM	[47]
9	2023	Similarity search+VS+MD	Drug design process	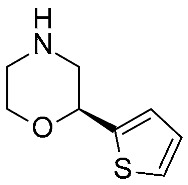 **17b**	0.405 μM	[48]
10	2023	Comparative docking+QSAR	Drug design process, selectivity profile elucidation	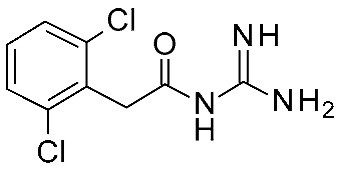 **Guanfacine**	20 nM	[49]
11	2024	In silico aided-drug design	SAR rationalization, drug design process	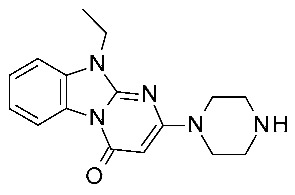 **18a**	526 nM	[50]

* **Ulotaront** was discovered through an in vivo phenotypic approach.

**Table 3:** List of the HMs produced in the context of drug discovery campaign towards *h/m*TAAR1 (*hTAAR1* in green, *m*TAAR1 in grey). References (Ref.), resolution (R), release date (R.D.), and percentage of identity (% Id) are reported.

Model n.	TAAR Models	First Published in Ref	Utilized in Ref (s)	Template	R (Å)	R.D.	Presence of Small Molecules/Ligand-Based HM	% Id. (BLAST)
1	*h*TAAR1	[40]	[33,40,42,43,44,86]	3PDS	3.50	2011	Irreversible agonist (co-crystallized)	31.60%
2	*h*TAAR1	[41]	[41]	2RH1	2.40	2007	Carazolol (partial inverse agonist) + ligand guided HM	31.60%
3	*h*TAAR1	[45]	[45]	HM by GPCRdb9 web site. Backbone of the TM helices: 3SN6 (prevalent template) loop coordinates: 6CM4, 4IAQ, 4UHR	3SN6: 3.2 6CM4: 2.87 4IAQ:2.80 4UHR: 2.60	3SN6: 2011 6CM4: 2018 4IAQ:2013 4UHR: 2015	3SN6: high affinity agonist (BI-167107) 6CM4: Risperidone (inverse agonist) 4IAQ: Dihydroergotamine (agonist) 4UHR: selective agonist CGS21680	31.60% (3SN6)
4	*h*TAAR1	Online source (AlphaFold, Structure ID Q96RJ0) [62,63] last updated in AlphaFold DB version 2022-11-01	[46,47,48]	Structure ID Q96RJ0	NA	NA	no	NA
5	*m*TAAR1	[27]	[27,28,42,43,86]	3PDS	3.50	2011	Irreversible agonist + ligand-based HM (T1AM)	31.48%

**Table 4:** List of the available PDB entries (PDB ID) containing *h*TAAR1 (in cyan) or *m*TAAR1 (in grey) data. This piece of information has been listed based on the resolution parameter (Å). The corresponding number of non-hydrogen atoms (n. of non-H atoms) and corresponding references (Ref.) are reported.

PDB ID	Ligand Name	Resolution (Å)	n. of Non-H Atoms	Ref.
8W88	**Ulotaront**	2.60	8307	[87]
8W87	**METH**	2.80	8228	[87]
8W8A	**RO5256390**	2.80	8260	[87]
8JLQ	**Fenoldopam**	2.84	9033	[90]
8WC8	**ZH8651**	2.90	8062	[91]
8W89	**β-PEA**	3.00	8230	[87]
8JLR	**A77636** (adamantane derivative)	3.00	8934	[90]
8JLP	**Ralmitaront**	3.23	7343	[90]
8JLN	**T1AM**	3.24	9117	[90]
8UHB	**RO5256390**	3.35	8417	[92]
8JSO	**D-AMPH**	3.40	9045	[90]
8WCA	**β-PEA**	3.48	8160	[91]
8JLO	**Ulotaront**	3.52	7343	[90]
8WC3	**Ulotaront**	3.00	8418	[91]
8WCC	**CHA**	3.04	2056	[91]
8JLJ	**T1AM**	3.10	8918	[90]
8WCB	**CHA**	3.10	7784	[91]
8WC7	**ZH8667**	3.10	8532	[91]
8WC4	**ZH8651**	3.10	8394	[91]
8WC9	**ZH8651**	3.20	8394	[91]
8WC6	**β-PEA**	3.20	9183	[91]
8JLK	**Ulotaront**	3.22	8824	[90]
8WC5	**TMA**	3.30	8511	[91]

**Table 5:** Effect of residue mutation in *h*TAAR1 in terms of activity impairment with respect to the maximum ligand-induced activation. NO (white): none. YES (dark green): the mutation strongly impairs or eliminates activity. PARTIAL (orange): the mutation partially diminishes activation. POOR (dark pink): the activation diminishment is poor. Blue: the mutation augments the ligand-induced activation. Grey cells: no data available.

	*h*TAAR1 Agonists					
Protein Mutants	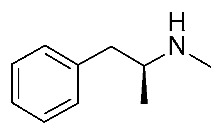 METH [87]	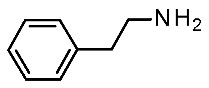 β-PEA [87]	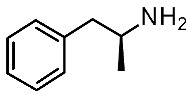 (*S*)-AMPH [87]	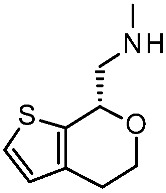 Ulotaront [87]	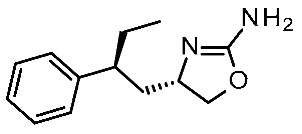 RO5256390 [87]	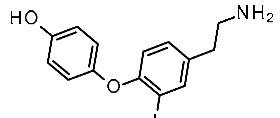 T1AM [87]
D103A	YES	YES	YES	YES	YES	YES
I104A	YES	YES	YES	YES	YES	YES
S107A	YES	YES	YES	YES	YES	YES
F186A	YES	YES	POOR	YES	YES	YES
T194A	YES	YES	-	YES	YES	YES
W264A	YES	YES	YES	YES	YES	YES
F267A	PARTIAL * (70%)	YES	POOR	PARTIAL * (56%)	YES	YES
Y294A	YES	YES	YES	YES	YES	YES
I290T	YES	YES	-	YES	YES	-
I290N	YES	YES	-	YES	YES	-
S80A	POOR * (84%)	PARTIAL (47%)	-	PARTIAL (53%)	PARTIAL (52%)	YES
R83A	YES	YES	-	YES	YES	YES
F185A	NO * (107%)	PARTIAL (52%)	-	PARTIAL (66%)	PARTIAL * (58%)	-
H99A	YES	YES	-	YES	YES	YES
S107C	YES	YES	-	YES	YES	-
S198A	ACTIVATION (150%)	ACTIVATION (129%)	-	NO (99%)	ACTIVATION (113%)	PARTIAL
S108A	-	-	-	-	-	YES
I290A	-	-	YES	-	-	-
F268A	-	-	PARTIAL (~50%)	-	-	YES
V184A	-	-	PARTIAL (~50%)	-	-	YES

* Such data exhibit a large standard deviation value.

**Table 6:** Effect of residue mutation in *m*TAAR1 in terms of activity impairment with respect to the maximum ligand-induced activation. NO (white): none. YES (dark green): the mutation strongly impairs or eliminates activity. PARTIAL (orange): the mutation partially diminishes activation. POOR (dark pink): the activation diminishment is poor. Grey cells: no data available.

*m*TAAR1 Agonists						
Protein Mutants	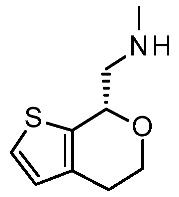 Ulotaront [90]	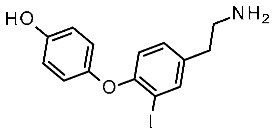 T1AM [90]	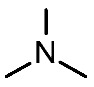 TMA [91]	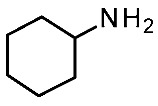 CHA [91]	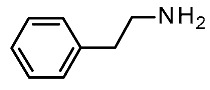 β-PEA [91]	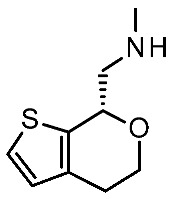 Ulotaront [91]
D102A	YES	YES	YES	YES	YES	YES
S106A	YES	YES	PARTIAL	NO	YES	PARTIAL
I103A	PARTIAL (~60%)	YES	POOR	PARTIAL	PARTIAL	PARTIAL
F185A	POOR *	YES	POOR	PARTIAL	PARTIAL	YES
W261A	YES	YES	YES	YES	YES	YES
F264A	YES	PARTIAL (~50%)	PARTIAL	PARTIAL	PARTIAL	PARTIAL
F265A	YES	YES	NO	PARTIAL	YES	PARTIAL
Y291A	-	YES	YES	NO *	YES	YES
S107A	-	POOR	-	-	-	-
P183A	-	POOR	POOR	NO	PARTIAL	YES
A193T	-	POOR	-	-	-	-
Y153A	-	-	NO	NO	YES	PARTIAL
S197A	-	PARTIAL (~50%)	NO	NO	NO	PARTIAL
Y287A	-	-	PARTIAL	PARTIAL	YES	PARTIAL

* Such data exhibit a large standard deviation value.

**Table 7:** Drug discovery studies focused on *m*TAAR5 antagonists, involving modelling techniques. The related references (Ref.) have been reported.

Entry	Year	Method of Discovery	Use of Structural Information	Proposed Hits	mTAAR5 IC_50_	Ref.
1	2016	VS on *m*TAAR5 HM	Prospective VS, selectivity rationalization	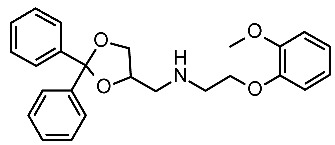 **19**	29 μM	[86]
				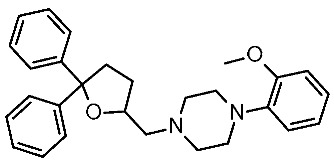 **20**	4.8 μM	
2	2022	VS on *m*TAAR HM	Prospective VS	**21**, **22** Chemical structure not shown	1.1 μM	[121]
3	2023	VS on *m*TAAR HM	Prospective VS	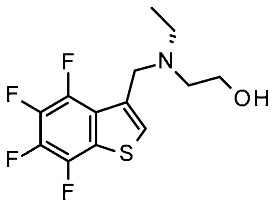 **23**	21 μM	[122]
				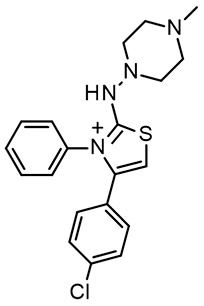 **24**	3.5 μM	
				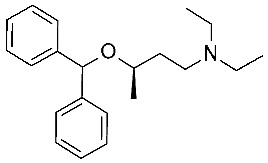 **25**	2.8 μM	

**Table 8:** List of the HMs produced in the context of drug discovery campaigns towards *h/m*TAAR5 (*h*TAAR5 in green, *m*TAAR5 in grey). References (Ref.), resolution (R), release date (R.D.), and percentage of identity (% Id) are reported.

Model n.	Modelled TAAR	Generated in Ref.	Utilized in Ref (s)	Template	R (Å)	R.D.	Presence of Small Molecules/Ligand-Based HM	% Id. (BLAST)
1	*h*TAAR5	[86]	[42,86]	3PDS	3.50	2011	Irreversible agonist	34.26%
2	*m*TAAR5	[86]	[42,86]	3PDS	3.50	2011	Irreversible agonist	33.89%
3	*m*TAAR5	[121]	[121]	6IBL	2.70	2019	Formoterol (agonist)	33.33%
4	*m*TAAR5	[122]	[122]	Main templates: 4GBR, 2Y03 ECL2: 5ZBH	4GBR: 3.99, 2Y03: 2.85, 5ZBH: 3.00	4GBR: 2012 2Y03: 2010 5ZBH: 2018	4GBR: S-Carazolol (inverse agonist) 2Y03: Isoprenaline (agonist) 5ZBH: BMS-193885 (antagonist)	4GBR: 33.89% 2Y03: 33.33% 5ZBH: 24.44%

**Table 9:** BLAST-p alignment results according to the BLOSUM62 matrix, using *h*TAAR5 as query. The proposed template is indicated via its PDB ID, name of the macromolecule, organism, BLAST total score, coverage of the sequence, and percentage of identity (% Id.) between the query and the template.

PDB ID	Description	Scientific Name	Total Score	Query Cover	% Id.
8ITF	Trace amine-associated receptor 9	*Mus musculus*	299	97%	46.45%
8PM2	Trace amine-associated receptor 7f	*Mus musculus*	321	96%	45.65%
8W87	Trace amine-associated receptor 1	*Homo sapiens*	251	95%	38.74%
8JLN	Trace amine-associated receptor 1	*Homo sapiens*	253	99%	38.33%
8JLJ	Trace amine-associated receptor 1	*Mus musculus*	247	99%	37.90%
6H7J	β-_1_ adrenergic receptor	*Meleagris gallopavo*	158	84%	37.72%
6IBL	β-_1_ adrenergic receptor	*Meleagris gallopavo*	158	84%	37.72%
2VT4	β-_1_ adrenergic receptor	*Meleagris gallopavo*	155	82%	37.41%
6TKO	β-_1_ adrenergic receptor	*Meleagris gallopavo*	159	82%	37.23%
2Y00	β-_1_ adrenergic receptor	*Meleagris gallopavo*	153	82%	37.14%
7JJO	β-_1_ adrenergic receptor	*Meleagris gallopavo*	152	81%	36.62%
5A8E	β-_1_ adrenergic receptor	*Meleagris gallopavo*	150	82%	36.43%
4BVN	β-_1_ adrenergic receptor	*Meleagris gallopavo*	149	82%	36.43%
7EJ0	α-_2A_ adrenergic receptor	*Homo sapiens*	160	82%	36.14%
7XT8	5-hydroxytryptamine receptor 4	*Homo sapiens*	157	80%	35.45%
4LDE	β-_2_ adrenergic receptor	*Homo sapiens*	139	77%	34.96%
5JQH	β-_2_ adrenergic receptor	*Homo sapiens*	139	77%	34.96%
4QKX	β-_2_ adrenergic receptor	*Homo sapiens*	137	77%	34.96%
8HN1	α-_1A_ adrenergic receptor	*Homo sapiens*	146	89%	34.85%
7BTS	β-_1_ adrenergic receptor	*Homo sapiens*	144	81%	34.78%
6MXT	β-_2_ adrenergic receptor	*Homo sapiens*	137	77%	34.59%
8THK	α-_1A_ adrenergic receptor	*Homo sapiens*	150	86%	34.54%
6KUY	α-_2A_ adrenergic receptor	*Homo sapiens*	161	85%	34.51%
2R4S	β-_2_ adrenergic receptor	*Homo sapiens*	132	77%	34.27%
2R4R	β-_2_ adrenergic receptor	*Homo sapiens*	131	77%	34.27%
4GBR	β-_2_ adrenergic receptor	*Homo sapiens*	136	77%	34.21%
7YS6	5-hydroxytryptamine receptor 6	*Homo sapiens*	134	83%	34.12%
6KUX	α-_2A_ adrenergic receptor	*Homo sapiens*	160	85%	34.07%
6WGT	5-hydroxytryptamine receptor 2A	*Homo sapiens*	134	82%	33.78%
3P0G	β-_2_ adrenergic receptor	*Homo sapiens*	147	78%	33.68%
2RH1	β-_2_ adrenergic receptor	*Homo sapiens*	147	78%	33.68%
7BZ2	β-_2_ adrenergic receptor	*Homo sapiens*	133	77%	33.46%
7DHI	β-_2_ adrenergic receptor	*Homo sapiens*	133	77%	33.46%
3SN6	β-_2_ adrenergic receptor	*Homo sapiens*	133	77%	33.45%
6NI3	β-_2_ adrenergic receptor	*Homo sapiens*	133	77%	33.45%
3D4S	β-_2_ adrenergic receptor	*Homo sapiens*	148	78%	33.16%
6PRZ	β-_2_ adrenergic receptor	*Homo sapiens*	148	77%	33.16%
**3PDS**	β-_2_ adrenergic receptor	***Homo sapiens***	**146**	**77%**	**33.16%**

**Table 10:** BLAST-p alignment results according to the BLOSUM62 matrix, using *m*TAAR5 as query. The proposed template is indicated via its PDB ID, name of the macromolecule, organism, BLAST total score, coverage of the sequence, and percentage of identity (% Id.) between the query and the template. TAARs as templates are highlighted in cyan.

PDB ID	Description	Organism	Total Score	Query Coverage	% Id.
8ITF	Trace amine-associated receptor 9	*Mus musculus*	305	97%	46.45%
8PM2	Trace amine-associated receptor 7f	*Mus musculus*	310	95%	44.24%
8WC3	Trace amine-associated receptor 1	*Mus musculus*	258	89%	42.44%
8JLJ	Trace amine-associated receptor 1	*Mus musculus*	260	89%	42.44%
8W87	Trace amine-associated receptor 1	*Homo sapiens*	259	94%	39.27%
8JLN	Trace amine-associated receptor 1	*Homo sapiens*	260	94%	39.27%
8UHB	Trace amine-associated receptor 1	*Homo sapiens*	258	96%	38.39%
6TKO	Beta-_1_ adrenergic receptor	*Meleagris gallopavo*	158	83%	37.46%
2Y00	Beta-_1_ adrenergic receptor	*Meleagris gallopavo*	155	83%	36.90%
7JJO	Beta-_1_ adrenergic receptor	*Meleagris gallopavo*	154	83%	36.90%
6H7J	Beta-_1_ adrenergic receptor	*Meleagris gallopavo*	159	84%	36.82%
2VT4	Beta-_1_ adrenergic receptor	*Meleagris gallopavo*	158	83%	36.81%
6WGT	5-hydroxytryptamine receptor 2A	*Homo sapiens*	108	86%	36.70%
6IBL	Beta-_1_ adrenergic receptor	*Meleagris gallopavo*	159	86%	36.67%

**Table 11:** BLAST-p alignment results for the *h*TAAR1 sequence considering only human GPCRs according to the BLOSUM62 matrix. The proposed reference GPCR is indicated via its PDB ID, name of the macromolecule, BLAST total score, coverage of the sequenceand percentage of identity (% Id.) between the query and the template.

PDB ID	Description	Total Score	Query Cover	% Id.
8W87	Trace amine-associated receptor 1	697	100%	100.00%
7WC4	5-hydroxytryptamine receptor 2A	67.8	23%	44.58%
7VOD	5-hydroxytryptamine receptor 2A	67.8	23%	44.58%
7XT8	5-hydroxytryptamine receptor 4	199	86%	37.42%
7CKY	D(1A) dopamine receptor	158	82%	33.55%
7CKX	D(1A) dopamine receptor	158	82%	33.55%
7CKW	D(1A) dopamine receptor	158	82%	33.55%
7F0T	D(1A) dopamine receptor	158	82%	33.55%
7JV5	D(1A) dopamine receptor	157	82%	33.55%
7B6W	Alpha-_1B_ adrenergic receptor	154	87%	32.78%
4GBR	Beta-_2_ adrenergic receptor	158	85%	32.65%
7C61	5-hydroxytryptamine receptor 1B	174	85%	32.44%
7BZ2	Beta-_2_ adrenergic receptor	167	85%	31.97%
7DHI	Beta-_2_ adrenergic receptor	167	85%	31.97%
7XTC	5-hydroxytryptamine receptor 7	138	82%	31.75%
6KR8	Beta-_2_ adrenergic receptor	170	86%	31.61%
2R4S	Beta-_2_ adrenergic receptor	164	85%	31.60%
2R4R	Beta-_2_ adrenergic receptor	163	85%	31.60%
3KJ6	Beta-_2_ adrenergic receptor	162	85%	31.60%
5V54	5-hydroxytryptamine receptor 1B	175	85%	31.58%
7YM8	alpha1A adrenergic receptor	165	78%	31.58%
7RAN	5-hydroxytryptamine receptor 2A	125	88%	31.29%
6LUQ	Chimera of D(2) dopamine receptor and Endolysin	169	84%	31.16%
7UL3	Histamine H2 receptor	139	89%	31.05%
6K42	Alpha-_2A_ adrenergic receptor	145	84%	31.03%
7EJ0	Alpha-_2A_ adrenergic receptor	167	88%	30.77%
5D5A	Beta-_2_ adrenergic receptor	175	78%	30.77%
7YS6	5-hydroxytryptamine receptor 6	144	85%	30.58%
8E9W	Muscarinic acetylcholine receptor M3	132	86%	30.57%
8E9Z	Muscarinic acetylcholine receptor M3	131	86%	30.57%
8E9Y	Muscarinic acetylcholine receptor M3	131	86%	30.57%
6G79	5-hydroxytryptamine receptor 1B	151	85%	30.43%
8JLZ	5-hydroxytryptamine receptor 6	147	85%	30.42%
7XTB	5-hydroxytryptamine receptor 6	146	85%	30.42%
5CXV	Muscarinic acetylcholine receptor M1	140	76%	30.27%
6KUW	Alpha-_2C_ adrenergic receptor	156	85%	30.26%
7YMJ	Alpha-_1A_ adrenergic receptor	127	87%	30.00%

**Table 12:** Conservation (†) of the binding-site residues of a set of *h*GPCR with respect to *h*TAAR1 are indicated in yellow. Otherwise, the mutated amino acids are listed. The dopamine receptor D_1_R, the α_1a_-ADR, α_1b_-ADR, the β_2_-ADR, the hystidine receptor type 2 (H2R), and muscarinic one type 3 have been reported.

Reference Proteins	*h*TAAR1 Binding Site																								
	T100	I104	V184	F186	D103	R83	V76	M77	S297	L72	Y294	W291	G293	I290	W264	F267	S107	S108	T271	F268	S198	T194	H99	S80	S190
5-HT_2A_R	I	V	L	D	†	T	†	†	†	†	†	†	†	V	†	†	†	T	N	†	†	G	W	†	†
D_1_R	V	†	S	L	†	A	†	†	†	V	†	†	†	V	†	†	†	T	N	†	†	S	W	K	†
α_1B_-ADR	A	V	†	E	†	L	†	L	†	†	†	†	†	L	†	†	C	T	L	†	†	S	W	†	Y
β_2_-ADR	†	V	F	T	†	H	†	V	†	M	†	†	†	N	†	†	V	T	N	†	†	S	W	G	Y
α_1A_-ADR	A	V	I	E	†	F	†	L	†	†	†	†	†	F	†	†	C	T	M	†	†	S	W	†	Y
H_2_R	†	V	†	V	†	Y	†	L	†	†	†	†	†	L	†	Y	C	T	F	†	T	D	Y	†	†
5-HT_6_R	†	V	L	A	†	N	†	†	†	V	†	†	†	T	†	†	C	†	N	†	T	A	W	A	F
M_3_R	L	C	I	L	†	Y	S	†	Y	I	†	†	†	Y	†	Y	†	N	V	N	G	†	W	F	I

**Table 13:** BLAST-p alignment results to the *h*TAAR5 sequence considering only human GPCRs according to the BLOSUM62 matrix. The proposed reference GPCR is indicated via its PDB ID, name of the macromolecule, BLAST total score, coverage of the sequence and percentage of identity (% Id.) between the query and the template.

PDB ID	Description	Total Score	Query Cover	% Id.
8W87	Trace amine-associated receptor 1	251	95%	38.74%
7EJ0	Alpha-_2A_ adrenergic receptor	160	82%	36.14%
7XT8	5-hydroxytryptamine receptor 4	157	80%	35.45%
6KUY	Alpha-_2A_ adrenergic receptor	161	85%	34.51%
2R4S	Beta-_2_ adrenergic receptor	132	77%	34.27%
2R4R	Beta-_2_ adrenergic receptor	131	77%	34.27%
4GBR	Beta-_2_ adrenergic receptor	136	77%	34.21%
7YS6	5-hydroxytryptamine receptor 6	134	83%	34.12%
6KUX	Alpha-_2A_ adrenergic receptor	160	85%	34.07%
5D5A	Beta-_2_ adrenergic receptor	147	78%	33.68%
7BZ2	Beta-_2_ adrenergic receptor	133	77%	33.46%
7DHI	Beta-_2_ adrenergic receptor	133	77%	33.46%
3KJ6	Beta-_2_ adrenergic receptor	132	77%	33.10%
6KR8	Beta-_2_ adrenergic receptor	136	82%	32.46%
7SRQ	5-hydroxytryptamine receptor 2B	114	78%	32.26%
7SRS	5-hydroxytryptamine receptor 2B	112	78%	32.26%
8JLZ	5-hydroxytryptamine receptor 6	128	83%	32.17%
7XTB	5-hydroxytryptamine receptor 6	128	83%	32.17%
6K42	Alpha-_2B_ adrenergic receptor	133	81%	31.97%
7B6W	Alpha-_1B_ adrenergic receptor	148	91%	31.78%
7UL3	Histamine H2 receptor	108	83%	31.58%
7YMJ	Alpha-_1A_ adrenergic receptor	123	89%	31.27%
8HDO	Adenosine A2b receptor	105	81%	31.14%
7YM8	Alpha-_1A_ adrenergic receptor	164	86%	30.77%
6KUW	Alpha-_2C_ adrenergic receptor	146	82%	30.60%
7C61	5-hydroxytryptamine receptor 1B	155	80%	30.33%
6LUQ	Chimera of D(2) dopamine receptor and Endolysin	150	79%	30.11%
8JSP	5-hydroxytryptamine receptor 1A	149	79%	30.00%

**Table 14:** Conservation (†) of the binding-site residues of a set of *h*GPCR with respect to *h*TAAR5 is reported in yellow. Otherwise, the mutated amino acids are listed.

Reference Proteins	*h*TAAR5 Binding Site																								
	L83	V87	L88	S91	R94	H110	T111	D114	T115	C118	L119	L194	L196	W200	N204	L207	W265	F268	T269	T272	I291	W292	A294	Y295	S298
α_2A_-ADR	V	†	I	†	N	Y	L	†	V	†	T	I	gap	†	S	S	†	†	F	Y	F	†	G	†	†
β_2_-ADR	M	†	V	G	H	W	†	†	V	V	T	F	T	Y	S	S	†	†	F	N	N	†	G	†	†
5-HT_6_R	V	†	M	A	N	W	†	†	V	†	S	†	A	F	S	T	†	†	F	N	T	†	G	†	†
H_2_R	†	†	†	†	Y	Y	†	†	V	†	T	V	V	Y	D	T	†	Y	F	F	L	†	G	†	†
α_1A_-ADR	†	†	†	†	F	W	A	†	V	†	T	I	E	Y	A	S	†	†	F	M	F	†	G	†	†
5-HT_1B_R	V	†	M	†	Y	W	L	†	I	†	T	I	Y	gap	T	A	†	†	F	S	T	†	G	†	†
5-HT_1A_R	V	†	†	A	Y	F	I	†	V	†	T	I	K	Y	T	A	†	†	F	A	N	†	G	†	†
D_1_R	V	†	M	K	A	W	V	†	I	S	T	S	†	Y	S	S	†	†	F	N	V	†	G	W	†

## Data Availability

Data sharing not applicable.

## List of Abbreviations

**β-PEA**	**β-phenylethylamine**
**T1AM**	3-iodothyronamine
**TMA**	trimethylamine
**CHA**	cyclohexylammonium ion
**METH**	methamphetamine
**AMPH**	amphetamine
**(Q)SAR**	(quantitative) structure-activity relationship
**HM**	homology model
**VS**	virtual screening
**HTS**	high throughput screening
**ROC-AUC**	(receiver operating characteristic-area under the curve)
**RMSD**	root mean square deviation
**AF**	AlphaFold
**β_2_-ADR**	β_2_-adrenoreceptor
**5-HT_6_R**	5-hydroxytryptamine receptor 6
**5-HT_2A_R**	5-hydroxytryptamine receptor 2A
**5-HT_2B_R**	5-hydroxytryptamine receptor 2B
**M_3_R**	muscarinic acetylcholine receptor
**D_1_R**	dopamine D1 receptor
**α_1A_-ADR**	α_1A_-adrenergic receptor
**α_1B_-ADR**	α _1B_-adrenergic receptor
**α_2A_-ADR**	α_2A_-adrenergic receptor
**H_2_R**	hystidine receptor type 2
**SEP-363856**	**ulotaront**
**RO-6889450**	**ralmitaront**
